# Changes in DNA methylation–based aging predicts brain damage and dementia and reflects life‐course cardiovascular risk

**DOI:** 10.1002/alz.71632

**Published:** 2026-06-27

**Authors:** Nigus Gebremedhin Asefa, Jorge Martinez Romero, Yi‐Han Hu, Zhiguang Li, Osorio Meirelles, Djassinabaye Mbangdadji, May A. Beydoun, Sigurdur Sigurdsson, Valborg Gudmundsdottir, Thor Aspelund, Vilmundur Gudnason, Lenore Launer

**Affiliations:** ^1^ Laboratory of Epidemiology and Population Sciences National Institute on Aging Baltimore Maryland USA; ^2^ Icelandic Heart Association Kopavogur Iceland; ^3^ Faculty of Medicine University of Iceland Reykjavik Iceland

**Keywords:** aging, biological aging, brain, cardiovascular health, dementia, DNA methylation, Life's Simple 7, life‐course, lifestyle factors, midlife

## Abstract

**INTRODUCTION:**

There is well‐established evidence showing an association between accelerated biological aging (BA) and brain pathology. However, it remains unclear whether dynamic change in BA during adulthood is directly associated with brain health or primarily reflects cumulative life‐course environmental and lifestyle exposures.

**METHODS:**

We analyzed data from the Age, Gene/Environment Susceptibility–Reykjavik Study (AGES‐RS; *n* = 2081), with assessments at midlife (≈50 [SD 6.3] years) and late‐life (baseline ≈76 [4.8]; follow‐up ≈81 [4.8] years). We identified individuals with substantial change in BA (measured by DunedinPACE) and examined associations with brain magnetic resonance imaging (MRI) and cognitive outcomes. Mediation analyses tested whether late‐life BA mediated associations between midlife cardiovascular health and later‐life brain health.

**RESULTS:**

Shifting to accelerated BA was associated with lower brain volumes and incident dementia. Associations with brain infarcts and cognitive function largely reflected cumulative life‐course exposure.

**DISCUSSION:**

These findings underscore the need for further investigation into the timing, reversibility, and pathways linking BA to brain health.

## Highlights

- In analyses using the full sample (average associations), higher baseline DunedinPACE was strongly associated with lower brain volumes, greater likelihood of infarcts, and poorer cognitive performance at follow‐up.
- After excluding participants with consistently *slow* or *fast* DunedinPACE trajectories over follow‐up, a shift toward accelerated DunedinPACE was associated with lower brain volumes; however, associations with infarcts, small vessel disease, and cognitive performance were less evident.
- Baseline DunedinPACE mediated the association between midlife cardiovascular health (Life's Simple 7 composite score) and follow‐up brain volumes, infarcts, and cognitive performance at follow‐up.
- Our findings underscore the potential of biological aging not only to identify individuals at elevated risk of brain damage when their pace of aging accelerates, but also to capture the cumulative effects of lifestyle and environmental exposures on brain aging.

## 1 BACKGROUND

Aging is a well‐established risk factor for functional and structural brain deterioration. However, considerable inter‐individual variability exists in these outcomes among people of the same chronological age. This variability may be better explained by differences in biological aging (BA) rather than chronological age alone. Over the past decade, researchers have developed DNA methylation (DNAm)–based algorithms to estimate BA.^1^, ^2^

Previous studies have shown that individuals whose BA exceeds their chronological age are more likely to develop white matter lesions (WMLs),^3^, ^4^ WM loss,^4^, ^5^ and cognitive decline.^5^, ^6^ Separately, a substantial body of evidence suggests that adverse experiences early in life are linked to poorer brain health, including cognitive decline in later life. For example, a large Japanese population‐based study showed that older adults who experienced three or more adverse childhood experiences have demonstrated an increased risk of dementia at a mean (SD) age of 73.5 (6.0) years.^7^ Extending beyond early‐life influences, these associations are also evident within mid‐ and late‐life. In the CARDIA study, poorer cardiovascular health in early midlife (mean age ≈45 years) predicted faster cognitive decline and greater accumulation of white matter hyperintensities over 10 years.^8^ Furthermore, metabolite profiles measured at ages 60–64 were associated with cognitive function in the British 1946 Birth Cohort.^9^ Together, these findings suggest that adverse effects of lifestyle and metabolic risk factors on brain health are detectable well before old age.

These findings may support that aging reflects multi‐system physiological decline, driven by processes such as metabolic dysfunction and inflammation that operate across tissues and organ systems.^10^ Accordingly, BA measures derived from epigenetic patterns of multiorgan system biomarkers—including immune, hepatic, cardiovascular, and renal function—such as DunedinPACE,^10^ may capture systemic processes that are closely linked to brain aging and neurodegeneration.^4^, ^11^

However, it remains unclear whether associations between BA and brain pathology^4^, ^5^, ^6^ in older adults primarily reflect dynamic changes in BA during late‐life or cumulative environmental and lifestyle exposures across the life‐course. This uncertainty highlights a gap in understanding whether late‐life improvements or deterioration in BA are associated with brain health, underscoring the need to examine longitudinal changes in BA in older adulthood as predictors of brain pathology.

In this prospective study, we aimed to examine whether individuals who transitioned toward an accelerated pace of BA over time were more likely to exhibit indicators of brain pathology at follow‐up. Furthermore, we investigated whether BA mediates the association between a composite score of midlife cardiovascular risk factors and late‐life brain deterioration. Finally, given that cognitive impairment and brain atrophy are early markers of dementia, we also conducted survival analyses to evaluate the association between pace of BA shift and risk of developing dementia, capturing a clinically meaningful endpoint. Consistent associations across these domains would strengthen the evidence for the influence of accelerated aging on brain health.

## 2 METHODS

Data are from the Age, Gene/Environment Susceptibility–Reykjavik Study (AGES‐RS). The AGES‐RS cohort originated from the Reykjavik Study, which was conducted between 1967 and 1996, by the Icelandic Heart Association to examine cardiovascular disease and risk factors among middle‐aged individuals. The AGES‐RS cohort and its measurements have been described extensively elsewhere.^12^ Briefly, AGES‐RS is a population‐based prospective cohort study of individuals born between 1907 and 1935 in the Reykjavik region, Iceland. Baseline data were collected between 2002 and 2006, from 5764 surviving members of the Reykjavik Study.^13^ Of these, 2602 individuals had DNA methylation data available. A second assessment was conducted between 2006 and 2011. During this phase, a total of 3316 participants underwent re‐examination after a median follow‐up of ≈5 years, with DNA methylation data available for 2081 individuals (Figure S1). All participants provided written informed consent, and ethical approval was granted by the Iceland National Bioethics Committee (VSN: 00–063).

**RESEARCH IN CONTEXT**

1. **Systematic reviews**: Our systematic review in PubMed identified multiple studies reporting associations between accelerated biological aging (BA), estimated using epigenetic clocks (including Horvath, GrimAge, PhenoAge, and DunedinPACE), and brain volume and cognitive function in older adults. However, it remains unclear whether changes in BA during older adulthood are independently associated with brain health, or whether they primarily reflect cumulative life‐course environmental and lifestyle exposures.
2. **Interpretation**: By focusing on subgroups with substantial 5‐year changes in the pace of BA (defined by mean ±SD), our findings suggest that accelerated BA is longitudinally associated with lower brain volumes over a median follow‐up of 5 years. However, associations with cognitive performance and infarcts were less evident within this follow‐up period. Our results also underscore the cumulative life‐course influence of midlife cardiovascular health on late‐life brain outcomes.
3. **Future directions**: Our findings warrant further investigation to address several key questions: (1) Does improvement in BA among older adults lead to better brain health outcomes? (2) Is dynamic change in BA more strongly associated with structural brain measures (e.g., gray matter volume) than with functional outcomes (e.g., processing speed)? (3) Does the absence of associations between BA and cognitive performance or infarcts in our analysis reflect the relatively short follow‐up period (median ≈5 years)? Alternatively, could it indicate that brain damage resulting from cumulative life‐course exposures is not reversible in older adults? (4) At what stage of the life‐course would interventions aimed at slowing BA be most effective in preventing or attenuating brain deterioration? (5) What molecular and epigenetic pathways link changes in DNA methylation to brain structural deterioration in older adults?

### 2.1 DNA methylation assay and estimation of the pace of aging

DNA methylation was assessed in whole blood from *n* = 2602 baseline and *n* = 2081 follow‐up participants, using the Illumina Infinium MethylEPIC v1 platform. Data preprocessing included bisulfite conversion, quantile normalization, and extensive quality control (QC) to remove low‐quality or cross‐reactive probes. After QC, a total of 819,802 autosomal cytosine–phosphate–guanine (CpG) probes were retained for analysis (see Supporting Information for details). DunedinPACE (DDPACE) scores were estimated using a publicly available algorithm at GitHub (https://github.com/danbelsky/DunedinPACE)^10^ (accessed on 01/30/2024). The DDPACE clock, scaled in years of BA per chronological year, is designed to capture the pace of aging.^10^ It quantifies the pace at which individuals are aging biologically relative to their chronological age. For comparison, we also calculated principal component (PC)–based epigenetic clocks, PC‐Horvath,^14^ PC‐GrimAge,^15^ PC‐Hannum,^1^ and PC‐PhenoAge,^16^ using R scripts provided by the Levine et al. lab.^17^

### 2.2 Phenotypic data

#### 2.2.1 Socio‐demographic data

Demographic information (sex and age) was collected at baseline assessment. Body mass index (BMI) was derived from measured weight and height (kg/m^2^). We collected smoking status (never, ex, and current) and education (elementary, high school, college, and university) using a self‐reported questionnaire.^18^ History of physical activity (PA), defined as moderate‐to‐vigorous PA over the past 12 months in hours per week, was also assessed via questionnaire.^18^, ^19^ Participants were instructed to fast overnight, after which serum cholesterol and fasting blood glucose levels were measured using an enzymatic colorimetric assay. Supine blood pressure was measured twice in fasting participants by a nurse using a sphygmomanometer, and the average of the two readings was recorded. Similar methods were used in the Reykjavik Study^13^ during midlife.

#### 2.2.2 MRI acquisition, brain tissue segmentation, and brain infarcts

Brain volumes and infarcts were assessed using magnetic resonance imaging (MRI) scans performed with a 1.5‐T Signa Twinspeed EXCITE system (GE Medical Systems, Waukesha, WI, USA) as part of the AGES‐RS. Details of MRI acquisition and brain volume measurements are discussed in detail by Muller et al. and Sigurdsson et al.,^20^, ^21^ summarized in the Supporting Information.

#### 2.2.3 Cognitive function

Details of the cognitive test battery and composite score construction are provided by Saczynski et al.^22^ and in the Supporting Information. Briefly, we used a standard battery of assessments, including the California Verbal Learning Test for memory, the Digit Symbol Substitution Test (DSST) for processing speed, and the Stroop Test for working memory. For each domain, we calculated composite scores by standardizing the raw scores from individual tests into *z*‐scores (mean = 0, SD = 1) and then averaging the *z*‐scores across tests within that domain. The global cognitive function score was then calculated as the average of these three domain scores.

#### 2.2.4 Dementia

Dementia status was ascertained through standard assessment tools (DSST and the Mini‐Mental State Examination [MMSE]), and with an additional more detailed neuropsychological test battery if individuals scored <18 on the DSST or <24 on the MMSE. A final consensus diagnosis was determined by a multidisciplinary panel comprising a geriatrician, neurologist, neuropsychologist, and neuroradiologist. Details of the diagnostic steps are provided by Sigurdsson et al.^21^ and in the Supporting Information.

### 2.3 Analytical sample and exclusion criteria

Figure S1 summarizes the timeline of data collection, analytical steps, and sample sizes for each analysis after exclusions. Analyses were conducted in three steps. In *Step 1*, association analyses included 1811 to 1901 participants across Models 1 and 2, depending on the outcome. *Step 2* (mediation analysis) included 1811 participants with complete data on midlife cardiovascular risk factors, baseline DDPACE, and follow‐up brain health indicators. In *Step 3*, survival analyses of dementia included 2381 participants in Model 1 and 1910 participants in Model 2.

### 2.4 Statistical analysis

#### 2.4.1 Descriptive analysis

We performed descriptive analyses; normally distributed continuous variables reported as means (SD), and non‐normal ones as medians (interquartile ranges [IQRs]). Categorical variables are presented as counts and percentages.

#### 2.4.2 Association analyses

##### 2.4.2.1 Association of DDPACE with brain health indicators

In *Step 1*, we applied two models: In *Model 1*, we examined prospective associations between baseline DDPACE and brain pathology outcomes at follow‐up (brain volume, brain infarcts, other small vessel disease (SVD) markers, and cognitive function) (Figure S1). Linear regression was used for continuous outcomes and logistic regression for binary outcomes, with results reported as β coefficients or odds ratios (ORs) and 95% confidence intervals (CIs). As an internal validation strategy, we performed 10‐fold cross‐validation (10‐CV) to assess the robustness of within‐cohort analyses, comparing model performance between in‐sample and cross‐validated models. In parallel, we performed similar analyses using the residuals of PC‐based measures, including PC‐Hannum, PC‐Horvath, PC‐GrimAge, and PC‐PhenoAge.

In the *Model 2*, we examined whether longitudinal changes in DDPACE—categorizing participants as *decelerators*, *average agers*, or *accelerators* (as described in our previous publication)^23^—were associated with the same outcomes. Briefly, individuals were first categorized at baseline as *slow* (≤1 SD below the mean), *average* (within ± 1 SD), or *fast* (≥1 SD above the mean) based on their DDPACE scores (Figure S2). The same thresholds were applied at follow‐up to reclassify individuals into these categories. Combining baseline and follow‐up classifications yielded nine possible transition groups (e.g., slow‐average, average‐fast, fast‐slow, etc.) (Figure S2). Because some trajectory groups contained very few or no participants, we collapsed them into three categories based on transitions in DDPACE: (1) *Decelerators* (improved aging status: *fast‐slow*, *average‐slow*, and *fast‐average*), (2) *Average agers* (average at both time points), and (3) *Accelerators* (worsened aging status: *slow‐average*, *average‐fast*, and *slow‐fast*). Our analyses focused specifically on individuals who exhibited changes (> mean ± 1 SD) in their pace of aging; therefore, participants with stable trajectories (*slow‐slow* and *fast‐fast*) were excluded prior to the shift‐based analysis, as these participants did not contribute to the transition‐focused analysis (Figure S2).

##### 2.4.2.2 Mediation analyses

In *Step 2*, we conducted mediation analyses to examine whether DDPACE mediated the association between midlife cardiovascular health (mean age 50, SD 6.3; Table S1) and later‐life brain volume, brain infarcts, and cognitive function (mean age 81; Figure S1). Lifestyle‐ and biometric‐related variable selection was based on the American Heart Association's Life's Simple 7 (LS7),^24^ which includes four health behaviors (smoking status, BMI, physical activity [or PA], and diet) and three biometric factors (blood pressure [BP], total cholesterol, and blood glucose levels). A higher LS7 score represents better cardiovascular health. As diet data were unavailable, we computed an adapted‐LS7 composite score based on a previously established algorithm.^19^ The mediation analysis was conducted using the mediation R package^25^ with 5000 bootstrap iterations. Robust standard errors were used (robustSE = TRUE) to ensure reliable inference.

##### 2.4.2.3 Survival analyses

In *Step 3*, we performed survival analyses to assess the hazard ratio (HR) of incident dementia. Two models were fit: one using baseline DDPACE as a continuous predictor (*Model 1*) and the other using DDPACE shift categories (*Model 2*) (Figure S1). Both models used a Cox proportional hazards model, and the proportional hazards assumption was tested and met. To account for the competing risk of death, we applied the Fine–Gray sub‐distribution hazard model, recognizing that dementia is a late‐life outcome and some individuals may die before its onset.

##### 2.4.2.4 Sensitivity analyses

We also conducted sensitivity analyses to assess the robustness of our findings. For the prospective association analyses between DDPACE and cognitive function, we (1) assessed potential effect modification by including an interaction term between DDPACE and apolipoprotein E ε4 (*APOE* ε4) carrier status (*ε3/ε4 or ε4/ε4*), and (2) re‐ran the models after excluding *ε2/ε4* carriers, the genotype of which includes alleles with opposing associations with dementia risk (*ε2* protective, *ε4* deleterious). In addition to the shift‐based analyses, we modeled DDPACE change (follow‐up minus baseline) as a continuous exposure and re‐examined its association with brain health outcomes in the full sample.

In the survival analyses of dementia, we performed three additional sensitivity analyses: (1) re‐estimating Cox proportional hazards models with a 2‐year lag; (2) repeating analyses after excluding ε2/ε4 carriers; and (3) evaluating potential effect modification by testing interactions between DDPACE and *APOE* ε4 carrier status, sex, smoking status, and educational level.

All models were adjusted for chronological age, sex, smoking status, educational level, estimated white blood cell composition, and assay batch. In addition, brain volume models were adjusted for *intracranial volume* to account for head size differences, whereas cognitive function and dementia models were further adjusted for *APOE* ε4 *status*, a known genetic risk factor for dementia.^26^ *APOE* ε4 status was determined using microplate array diagonal gel electrophoresis.^27^ For ease of comparison, DDPACE (in models where it was used as a continuous variable), brain volume, and cognitive function variables were scaled to SD units, and statistical significance was determined using a two‐sided *p*‐value threshold of *p *< 0.05. Analyses were conducted in R, version 4.5.1.

## 3 RESULTS

### 3.1 Descriptive analysis

The mean (SD) age was 75.5 (4.84) at baseline and 80.7 (4.84) years at follow‐up. There was a modest but statistically significant increase in DDPACE, from 1.10 at baseline to 1.13 at follow‐up (paired *T*‐test, *p* < 2.16 × 10^−16^) (Table 1). There were fewer current smokers and more ex‐smokers at follow‐up. Brain volumetric measures showed a clear pattern of decline, including reductions in white matter (WM) and gray matter (GM), total brain volume (TBV), and an increase in WM lesions (WMLs). Cognitive performance worsened across memory, processing speed, and working memory, reflecting decline at follow‐up assessment.

**TABLE 1:** Characteristics of study participants, including sociodemographic factors, DDPACE, and other variables, by time of data collection.

Variables	Baseline (2002 to 2006), *n* = 2081	Follow‐up (2006 to 2011), *n* = 2081	*p*‐value
**Age in years, mean (SD)**	75.51 (4.84)	80.71 (4.84)	< 0.001
**Sex, F, *n* (%)**	1198 (57.6)	1198 (57.6)	NA
**DDPACE scores, mean (SD)**	1.10 (0.11)	1.13 (0.12)	< 0.001
**BMI, kg/m^2^, mean (SD)**	27.25 (4.25)	26.74 (4.43)	< 0.001
**Smoking status, *n* (%)**			
Never	895 (43.1)	848 (41.6)	1.42E‐02
Ex	952 (45.9)	1010 (49.6)	
Current	229 (11.0)	180 (8.8)	
**Educational level, *n* (%)**			
Elementary	441 (21.3)	441 (21.3)	1.00E+00
High school	1045 (50.5)	1045 (50.5)	
College	335 (16.2)	335 (16.2)	
University	250 (12.1)	250 (12.1)	
***APOE* genotype, *n* (%)**			
ε2/ε2	8 (0.4)	8 (0.4)	NA
ε2/ε3	192 (9.2)	192 (9.2)	
ε2/ε4	39 (1.9)	39 (1.9)	
ε3/ε3	1307 (62.9)	1307 (62.9)	
ε3/ε4	489 (23.5)	489 (23.5)	
ε4/ε4	43 (2.1)	43 (2.1)	
**Brain volume** **^♀^**			
WM, mean (SD)	0.26 (0.02)	0.25 (0.02)	< 0.001
WML, median (IQR)	0.008 (0.01)	0.011 (0.014)	< 0.001
GM, mean (SD)	0.46 (0.03)	0.45 (0.03)	< 0.001
TBV, mean (SD)	0.73 (0.04)	0.71 (0.04)	< 0.001
**Cognitive function** ^*^			
Memory, mean (SD)	−0.001 (0.92)	−0.29 (1.04)	< 0.001
Speed, mean (SD)	0.02 (0.75)	−0.33 (0.94)	< 0.001
Working, mean (SD)	0.01 (0.78)	−0.26 (0.87)	< 0.001
Global cognitive function, mean (SD)	0.02 (0.66)	−0.27 (0.78)	< 0.001
**Brain infarcts**			
Any infarcts, Yes, *n* (%)	587/2,081 (31.8)	401/1,494 (26.8)^±^	NA
Cortical, Yes, *n* (%)	210/2,081 (11.4)	157/1,871 (8.4) ^±^	
Subcortical, Yes, *n* (%)	148/2,081 (8.0)	91/1,933 (4.7) ^±^	
Cerebral, Yes, *n* (%)	395/2,081 (21.4)	244/1,686 (14.5) ^±^	
Any SVD, Yes, *n* (%)	561/2,081 (30.5)	404/1,520 (26.6) ^±^	
EPVS, Yes, *n* (%)	299/2,081 (16.3)	65/1,782 (3.6) ^±^	
Microbleeding, Yes, *n* (%)	323/2,081 (17.6)	358/1,758 (20.4) ^±^	

Abbreviations: *APOE* ε4, apolipoprotein E gene status; BMI, body mass index; DDPACE, DunedinPACE scores; EPVS, enlarged perivascular space; GMV, gray matter volume; IQR, interquartile range; SD, standard deviation; SVD, small vessel disease; TBV, total brain volume; WM, white matter volume; WML, white matter lesion.

^♀^ We used relative measures of GM, WM, and WML, each calculated by dividing the respective volume by the intracranial volume (head size); as such, these variables are unitless.

* Memory, processing speed, and working memory were derived as averages of standardized (z‐scored) composites from multiple test tools (see *Supporting Information*).

^±^ Incident infarcts at follow‐up were defined among participants free of infarcts at baseline*. *.

In the DDPACE shifting group classification (Figure S2), chronological age differed only marginally across groups (*p* = 0.05) (Table 2). Participants in the consistent fast–fast group who were excluded from shift‐based analyses (Figure S2), showed less favorable baseline profiles, including higher WMLs, lower GM and TBV, and poorer cognitive performance (all *p*’s < 0.01; Table 2). In contrast, the consistent slow–slow group, also excluded, exhibited more favorable cognitive performance. The fast–fast group also had a higher proportion of male participants, more current/former smokers, and a greater prevalence of baseline infarcts (Table 2).

**TABLE 2:** Characteristics of study participants, including sociodemographic factors and other variables at baseline, by DunedinPACE shift categories.

Variable		Included in the shift‐based analyses (*n* = 1664) **^¥^**			Excluded from the shift‐based analyses (417) **^¥^**		
Socia‐demography and lifestyle	Overall	Decelerator	Average	Accelerator	Consistent slow–slow	Consistent fast–fast	*p*
***N***	2081	125	1136	403	151	266	
Age in years, mean (SD)	75.51 (4.84)	75.60 (5.06)	75.39 (4.74)	75.90 (5.01)	74.67 (4.76)	75.89 (4.91)	0.050
Sex, *n* (%)							
F	1198 (57.6)	74 (59.2)	654 (57.6)	240 (59.6)	119 (78.8)	111 (41.7)	< 0.001
M	883 (42.4)	51 (40.8)	482 (42.4)	163 (40.4)	32 (21.2)	155 (58.3)	
Smoking status, *n* (%)							
Never	895 (43.1)	55 (44.0)	499 (44.0)	190 (47.3)	93 (61.6)	58 (21.9)	< 0.001
Ex	952 (45.9)	50 (40.0)	534 (47.1)	175 (43.5)	55 (36.4)	138 (52.1)	
Current	229 (11.0)	20 (16.0)	100 (8.8)	37 (9.2)	3 (2.0)	69 (26.0)	
Educational level, *n* (%)							
Elementary	441 (21.3)	31 (24.8)	243 (21.5)	76 (18.9)	28 (18.5)	63 (23.8)	0.083
High school	1045 (50.5)	57 (45.6)	571 (50.6)	203 (50.5)	70 (46.4)	144 (54.3)	
College	335 (16.2)	21 (16.8)	180 (16.0)	73 (18.2)	36 (23.8)	25 (9.4)	
University	250 (12.1)	16 (12.8)	134 (11.9)	50 (12.4)	17 (11.3)	33 (12.5)	
**Pace of aging**							
DDPACE score, mean (SD)	1.10 (0.11)	1.15 (0.11)	1.08 (0.06)	1.07 (0.10)	0.91 (0.04)	1.28 (0.06)	< 0.001
**APOE4 carrier status,** ε3/ε4 or ε4/ε4, *n* (%)							
No	1541 (74.4)	93 (75.0)	838 (74.2)	300 (74.6)	114 (75.5)	196 (74.2)	0.997
Yes	529 (25.6)	31 (25.0)	291 (25.8)	102 (25.4)	37 (24.5)	68 (25.8)	
**Brain volume** ^♀^							
WM, mean (SD) of proportion	0.26 (0.02)	0.26 (0.02)	0.26 (0.02)	0.26 (0.02)	0.27 (0.02)	0.26 (0.02)	< 0.001
logWML, mean (SD)	−6.96 (1.23)	−6.94 (1.20)	−7.00 (1.25)	−6.94 (1.23)	−7.21 (1.15)	−6.69 (1.18)	0.001
GM, mean (SD) of proportion	0.46 (0.03)	0.46 (0.03)	0.46 (0.03)	0.46 (0.03)	0.46 (0.03)	0.45 (0.03)	< 0.001
TBV, mean (SD) of proportion	0.73 (0.04)	0.73 (0.04)	0.73 (0.04)	0.73 (0.03)	0.74 (0.03)	0.72 (0.04)	< 0.001
**Cognitive function** ^*^							
Memory, mean (SD) of *z*‐scores	0.00 (0.92)	0.03 (0.87)	0.03 (0.92)	−0.03 (0.94)	0.27 (0.89)	−0.26 (0.90)	< 0.001
Processing speed, mean (SD) of *z*‐scores	0.02 (0.76)	0.08 (0.75)	0.01 (0.75)	0.10 (0.74)	0.22 (0.73)	−0.18 (0.78)	< 0.001
Working, mean (SD) of *z*‐scores	0.01 (0.78)	−0.03 (0.85)	0.03 (0.77)	0.06 (0.77)	0.06 (0.75)	−0.16 (0.79)	0.006
Global cognitive function, mean (SD) of *z*‐scores	0.02 (0.66)	0.04 (0.68)	0.04 (0.65)	0.04 (0.65)	0.19 (0.65)	−0.20 (0.69)	< 0.001
**Cerebrovascular biometrics**							
Any brain infarcts, *n* (%)							
No	1258 (68.2)	71 (65.7)	708 (70.4)	244 (68.2)	98 (70.0)	137 (58.5)	0.012
Yes	587 (31.8)	37 (34.3)	297 (29.6)	114 (31.8)	42 (30.0)	97 (41.5)	
Any SVD biomarker, n (%)							
No	1278 (69.5)	71 (65.7)	708 (70.6)	244 (68.7)	102 (72.9)	153 (65.7)	0.442
Yes	561 (30.5)	37 (34.3)	295 (29.4)	111 (31.3)	38 (27.1)	80 (34.3)	

Abbreviations: DDPACE, DunedinPACE scores; GM, gray matter; logWML, log‐transformed white matter lesion; *p*, *p*‐value; SD, standard deviation; SVD, small vessel disease; TBV, total brain volume; WM, white matter.

^¥^ At each assessment, participants were categorized as slow, average, or fast agers according to the distribution of DDPACE scores, using thresholds based on the baseline mean and SD and applied consistently at both time points. Transitions in aging pace from baseline to follow‐up were then used to classify individuals as decelerators (e.g., fast at baseline and slow or average at follow‐up), average agers (remaining within the mean ± SD range), or accelerators (e.g., shifting from slow or average at baseline to fast at follow‐up). Individuals consistently on *slow*–*slow* or *fast*–*fast* were excluded from the downstream analyses.

^♀^ We used relative measures of GM, WM, and WML, each calculated by dividing the respective volume by the intracranial volume (head size); as such, these variables are unitless.

* Memory, processing speed, and working memory were derived as averages of standardized (z‐scored) composites from multiple test tools (see *Supporting Information*).

In *Model 1* of the survival analysis, there were 603 incident dementia cases (603/2,381), with a median follow‐up of 10.4 years (IQR: 4.2) from baseline to study end (Table 3). In *Model 2*, a total of 11 incident dementia cases were observed among decelerators (11/111) and 64 among accelerators (64/375), with a median follow‐up of 5.6 years (IQR: 2.1) from follow‐up visit to study end (Table 3).

**TABLE 3:** Association between DDPACE as a predictor and hazard ratio of dementia at the end of study.

				Before adjusting for baseline DDPACE		Adjusted for baseline DDAPCE		Adjusted for competing risk of mortality	
Variable	New cases/No. of participants at risk	Median (IQR) follow‐up time in years	Incidence rates per 1,000 person‐year	HR (95% CI)	*p*	HR (95% CI)	*P*	HR (95% CI)	*P*
**Model 1**									
Per 1 SD increase of baseline DDPACE scores, *n* = 2381	603/2,381	10.4 (4.2)^¥^	26.1 (24.1–28.2)	**1.21 (1.07–1.36)**	**2.13E–03**	NA	–	**1.17 (1.03 – 1.32)**	**1.2E–02**
**Model 2**								**Adjusted for both competing risk of mortality and baseline DDPACE**	
DDPACE shift, *n* = 1,534									
Average	129/1,048	5.6 (2.1)^♀^	22.0 (18.2–25.8)	Reference		Reference		Reference	
Decelerators	11/111		18.6 (7.6–29.6)	0.88 (0.47–1.67)	7.01E–01	0.91 (0.48–1.72)	7.65E– 01	0.88 (0.47–1.65)	6.8E–01
Accelerators	64/375		33.3 (25.1–41.4)	**1.66 (1.21–2.28)**	**1.67E–03**	**1.65 (1.20–2.27)**	**2.17E– 03**	**1.48 (1.06–2.07)**	**2.0E–02**

*Note*: All models were adjusted for chronological age, sex, smoking status, educational level, estimated white blood cell composition, APOE4 status, and assay batch.

***Model 1***: continuous DDPACE as predictor; ***Model 2***: categorical DDPACE shifts as predictor.

Abbreviations: 95% CI lower, lower 95% confidence interval; 95% CI upper, upper 95% confidence interval; *APOE* ε4, apolipoprotein E4 carrier status; DDPACE, DunedinPACE; HR, hazard ratio; IQR, interquartile range; *p*, *p*‐value; SD, standard deviation.

^¥^ Follow‐up period for incident dementia spanned from ***baseline*** (2002–2006) to end of study (2011–2015).

^♀^ Follow‐up period for incident dementia spanned from ***follow‐up*** (2006–2011) to end of study (2011–2015).

### 3.2 Prospective association analysis

**FIGURE 1:**
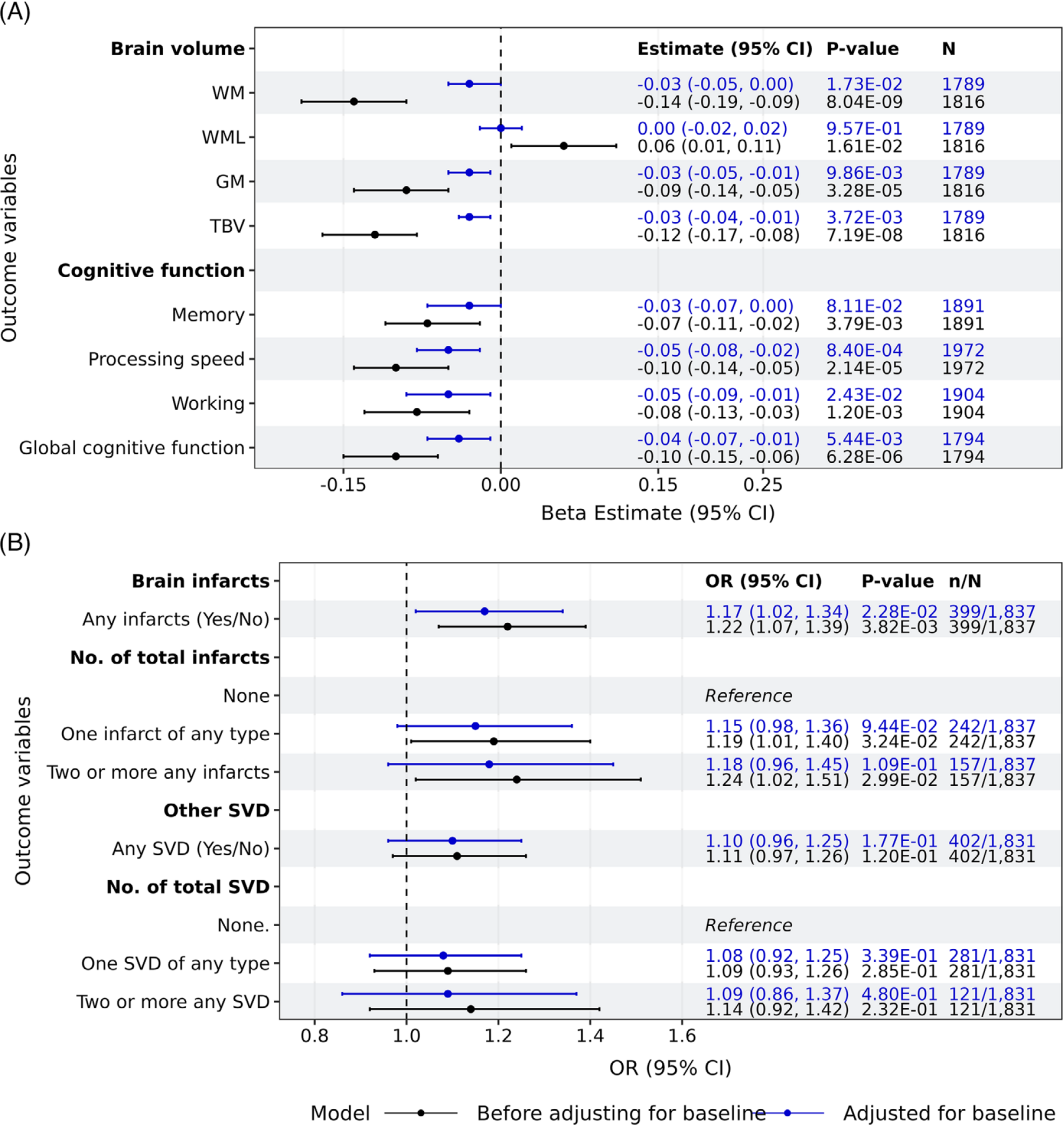
Prospective associations of baseline DDPACE with brain structure and cognitive function (A), and cerebrovascular outcomes (B) at follow‐up, before and after adjusting for baseline values of respective outcomes. This figure shows the prospective associations of baseline DDPACE with (A) brain volume and cognitive function outcomes and (B) brain infarcts and other SVD at follow‐up, and before and after adjustment for baseline values of the respective outcomes. Linear and logistic regression models were used, respectively, in A and B, and all models were adjusted for chronological age, sex, smoking status, educational level, estimated white blood cell composition, and assay batch. Brain volume models were additionally adjusted for intracranial volume, and cognitive function models were further adjusted for *APOE* ε4 status. In A, beta coefficients represent the change in brain volume or cognitive function outcomes (in SD units) per 1 SD increase in DDPACE scores; WML volume was log2‐transformed due to non‐normal distribution. In B, ORs represent the odds of having brain infarcts or other SVD per 1 SD increase in DDPACE scores. Values are shown before and after adjustment for baseline outcome values. APOE, apolipoprotein E; CI, confidence interval; DDPACE, Dunedin Pace of Aging Calculated from the Epigenome; GM, gray matter; n/N, number of outcome events over the total sample size; OR, odds ratio; SD, standard deviation; SVD, small vessel disease; TBV, total brain volume; WM, white matter; WML, white matter lesion.

Figure 1 summarizes the results of *Model 1* association analyses before and after adjustment for baseline brain outcomes. Individuals with higher baseline DDPACE were more likely to have lower volumes of WM (per 1 SD increase in DDPACE, β = −0.14, 95% CI: −0.19 to −0.09, *p* = 8.04×10^−09^), GM (β = −0.09, 95% CI: −0.14 to −0.05, *p* = 3.28×10^−^^5^), and TBV (β = −0.12, 95% CI: −0.17 to −0.08, *p* = 7.19×10^−^^8^), and higher WML volume (β = 0.06, 95% CI: 0.01–0.11, *p* = 1.61×10^−^^2^) (Figure 1A). After further adjusting for baseline brain volumes, the associations with WM, GM, and TBV remained significant. Similarly, higher baseline DDPACE was associated with poorer performance across all four cognitive domains (β ranging from −0.10 to −0.08, *all p*’s < 0.01). After further adjustment for baseline cognitive scores, these differences remained significant. No significant interaction was observed between *APOE* ε4 status and baseline DDPACE in predicting cognitive function outcomes at follow‐up (Table S2A). Similarly, after excluding *APOE* ε2/ε4 carriers, the associations between baseline DDPACE and cognitive function outcomes at follow‐up remained essentially unchanged (Figure 1A, Table S2B).

Individuals with higher baseline DDPACE were more likely to have any brain infarcts (per 1 SD increase in DDPACE, OR = 1.22, 95% CI: 1.07–1.39, *p* = 3.82×10^−^^03^) (Figure 1B). In a multinomial logistic model, higher baseline DDPACE was also associated with increased odds of having one infarct (OR = 1.19, 95% CI: 1.01–1.40, *p* *= *3.24×10^−02^) and two or more infarcts (OR = 1.24, 95% CI: 1.02–1.51, *p *= 2.99×10^−02^), compared to having no infarcts. These associations remained statistically significant after adjusting for baseline infarcts status, although the effect sizes were slightly attenuated (Figure 1B). Figure S3 presents the results for each specific type of brain infarct and other small vessel disease.

After applying 10‐fold cross‐validation, also adjusted for baseline values, the results showed consistent effect estimates and model fit compared to the original models, with minimal changes in R^2^ for continuous outcomes and a modest reduction in area under the curve (AUC) for infarct and other SVD‐related outcomes (Table S3). Furthermore, results from the DDPACE change analyses were consistent with the prospective findings, with a positive change in DDPACE (follow‐up minus baseline, full sample) associated with lower brain volume, poorer cognitive performance, and more WMLs and brain infarcts at follow‐up (Table S4).

Consistent with findings from DDPACE, higher values of second‐generation epigenetic clocks, PhenoAge and GrimAge, were associated with lower brain volume and poorer cognitive performance before adjusting for baseline values of the respective outcomes. After adjustment, associations of PC‐Horvath, PC‐Hannum, and PC‐PhenoAge clocks with brain health domains were no longer significant. In contrast, PC‐GrimAge remained significantly associated with brain volume and cognitive function outcomes (Table S5A). Associations of brain infarcts with PC‐Horvath, PC‐Hannum, and PC‐PhenoAge clocks were not statistically significant, except for GrimAge (Table S5B).

### 3.3 DDPACE shift analysis

**FIGURE 2:**
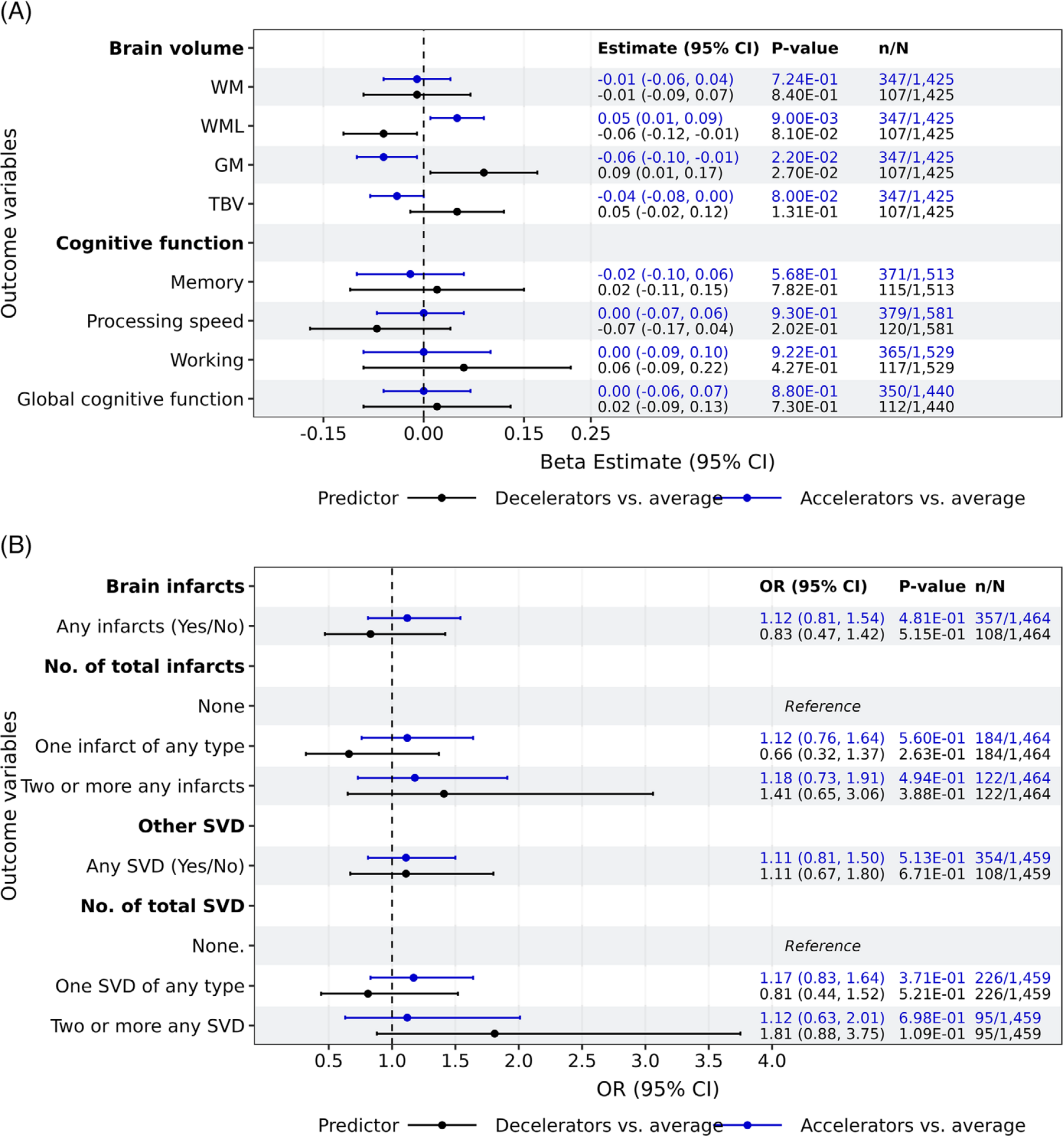
Associations of DDPACE shifts with brain structure and cognitive function (A), and cerebrovascular outcomes (B) at follow‐up. This figure shows the associations of shifts in DDPACE (average agers [reference], decelerators, and accelerators) with (A) brain volume and cognitive function outcomes and (B) brain infarcts and other small vessel disease (SVD) at follow‐up. Effect estimates compare decelerators versus average agers (black) and accelerators versus average agers (blue). All models were adjusted for chronological age, sex, smoking status, educational level, estimated white blood cell composition, assay batch, and baseline values of the respective outcome variables. Brain volume models were additionally adjusted for intracranial volume, and cognitive function models were further adjusted for *APOE* ε4 status. APOE, apolipoprotein E; CI, confidence interval; DDPACE, Dunedin Pace of Aging Calculated from the Epigenome; GM, gray matter; n/N, number of outcome events or number of accelerators (blue) and decelerators (black) over the total sample size for each model; OR, odds ratio; SVD, small vessel disease; TBV, total brain volume; WM, white matter; WML, white matter lesion.

In the shift‐based association analyses, those showing a decline in the DDPACE score (decelerators) had a significantly greater GM volume (β = 0.09; 95% CI: 0.01–0.17; *p* = 2.70 × 10^−02^) and a non‐significant trend toward lower WML volume (β = −0.06; 95% CI: −0.12 to 0.01; *p* = 8.10 × 10^−02^) compared to average agers at follow‐up (Figure 2A). In contrast, individuals who shifted to an accelerated pace of aging on the DDPACE scale (accelerators) had a higher WML load (β = 0.05; 95% CI: 0.01–0.09, *p* = 9.0 × 10^−03^), but lower GM volume (β = −0.06; 95% CI: −0.10 to −0.01, *p *= 2.2×10^−^^02^), compared to average agers at follow‐up (Figure 2A). No significant associations were found between shifts in DDPACE and cognitive function (Figure 2A), brain infarcts (Figure 2B), or other SVD (Figure 2B).

### 3.4 Mediation analysis

**FIGURE 3:**
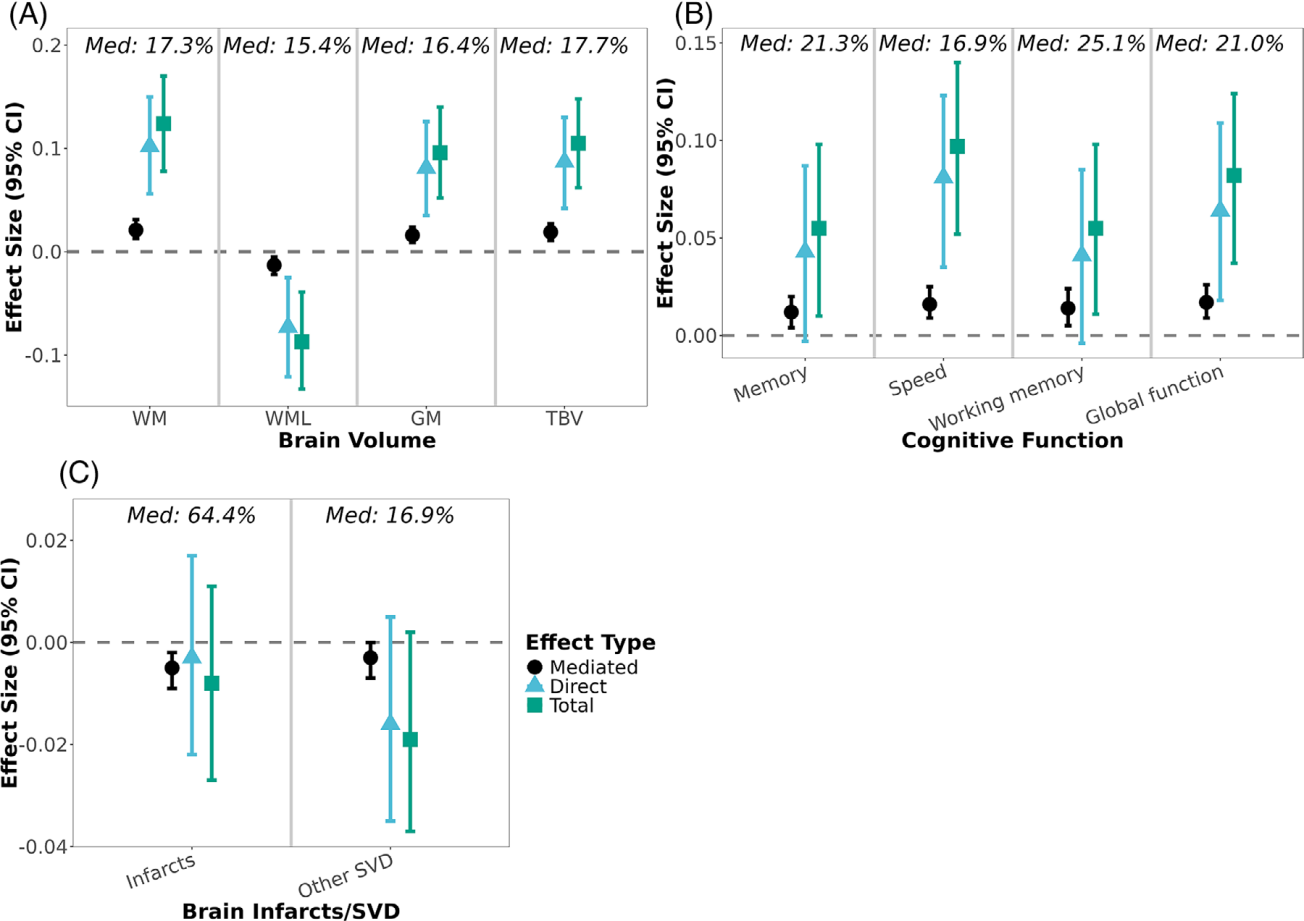
Mediation by baseline DDPACE in the association between midlife composite cardiovascular health score (adapted Life's Simple 7) and late‐life brain structures (A), cognitive function (B), and brain infarcts or SVD (C) at follow‐up. All models were adjusted for chronological age in midlife, sex, educational level, estimated white blood cell composition, and assay batch. DDPACE, DunedinPACE scores; CI, confidence interval; WM, white matter; WML, white matter lesion; GM, gray matter; TBV, total brain volume; LS7, adapted Life's Simple 7; SVD, small vessel disease; Med, proportion of mediation; Confidence interval’ *p*: *p*‐value.

DDPACE significantly mediated the association between midlife cardiovascular health (adapted‐LS7 composite score; measured at mean age ≈50 years) and late‐life brain volume measures (mean age ≈81 years), explaining 15.4% to 17.7% of the total estimates on WM, WML, GM, and TBV (all *p*’s < 0.001) (Figure 3A). Similarly, DDPACE also mediated the association between midlife adapted‐LS7 score and late‐life cognitive function, explaining 16.9% to 25% of the observed association (*p* < 0.001) (Figure 3B), and 16.9%–64.4% of the association with infarcts (Figure 3C).

### 3.5 Survival analysis of dementia incidence

Dementia‐free survival declined over time (Figure S4A). A 1 SD increase in DDPACE was associated with a 21% higher HR of dementia (HR = 1.21; 95% CI: 1.07–1.36; *p* = 2.13×10^−^^3^) (Table 3, Model 1). This association was slightly reduced (HR = 1.21 vs HR = 1.17) after accounting for the competing risk of death. The likelihood ratio test comparing the restricted cubic spline (knots = 4) model (non‐linear) to a linear model was not significant (χ^2^ = 2.22, *DF* = 3, *P* = 5.29E^−01^).

In Model 2, the median follow‐up time was 5.6 years (IQR: 2.1), with 129 new dementia cases among 1048 participants in the average group, 11 cases among 111 decelerators, and 64 cases among 375 accelerators. Dementia‐free survival probabilities differed significantly across DDPACE shift groups (*p* = 8.7 × 10^−^^3^), with accelerators showing a lower likelihood of remaining dementia‐free during follow‐up (Figure S4B). Compared to average agers, accelerators had a 66% higher risk of developing dementia during the follow‐up period (HR = 1.66; 95% CI: 1.21–2.28; *p* = 1.67×10^−^^3^). This association was similar when adjusting for baseline DDPACE (HR = 1.65; 95% CI: 1.20–2.27; *p* = 2.17×10^−^^3^) and persisted after further accounting for the competing risk of death (HR = 1.48; 95% CI: 1.06–2.07; *p* = 2.0×10^−^^2^) (Table 3, Model 2). The hazard ratio did not differ significantly between decelerators and average agers.

Sensitivity analyses using a 2‐year lag‐time and excluding individuals with the *APOE* ε2/ε4 genotype yielded results consistent with the original full‐sample analysis (both *p*’s < 0.01; Table S6 vs Table 3). Interaction analyses revealed no significant effect modification of the association between DDPACE and dementia incidence by *APOE* ε4, sex, smoking status, or educational level (Table S6).

## 4 DISCUSSION

In this study, we demonstrated that older individuals who had higher DDPACE at baseline had lower brain volumes, poorer cognitive function, and higher odds of brain infarcts over a median follow‐up of ≈5 years. Delta‐based and internal validation analyses showed consistent findings. In the shift‐based analyses, shifts toward either a decelerated or accelerated pace of aging were associated with brain volumes, but showed no significant associations with cognitive performance or brain infarcts. On the other hand, DDPACE mediated the association of midlife (mean age ≈50 years) composite cardiovascular health scores with brain deterioration and cognitive function in late life (mean age ≈81 years). From a clinical perspective, individuals classified as accelerators exhibited an increased hazard of developing dementia, independent of baseline DDPACE and accounting for the competing risk of all‐cause mortality.

When we employed the same analytical approach, our findings were in line with a previous study^4^ that reported consistent inverse associations of DDPACE with TBV, hippocampal volume, and mean cortical thickness, and a positive association with WML volume across three independent cohorts: the Dunedin Study (mean age 45 years; single‐year birth cohort), the ADNI cohort; mean age 75.4 years, range 55.0–95.6), and the FHS‐Offspring Cohort (mean age 63.8 years, range 40–82). Other studies with smaller study samples have also reported associations between GrimAge and WML,^3^, ^28^ the Horvath clock and WM,^29^ the DNAmCortical clock and WM,^29^ the Horvath and Hannum clocks and WML in African Americans,^30^ and the Horvath clock with both WM and GM in African teenagers.^31^ Our findings are also consistent with studies reporting associations between GrimAge and cortical atrophy and brain infarcts,^3^, ^4^, ^32^ as well as between DDPACE and multiple cognitive measures, including Digit Symbol, verbal fluency, and global cognitive scores, in both midlife and older adults.^33^, ^34^, ^35^

If these reported associations were robust, we would expect individuals who transitioned to an accelerated pace of aging to exhibit poorer brain health indicators, while those who shifted toward a decelerated pace would show better outcomes across all domains. However, in *Model 2*, where individuals with a consistently fast–fast or slow–slow pace of aging over time were excluded, focusing instead on those who experienced substantial transitions, the associations largely disappeared, except for brain volume traits (Figure 2). This raises an important question: why did the association of DDPACE with the other brain health indicators (cognitive function, infarcts, and other biomarkers of SVD) lose significance while the association with brain volume remained significant, despite using similar analytical approaches?

Several plausible explanations may account for this. First, although creating DDPACE transition groups reduces sample size, this alone is unlikely to explain the loss of significance: the brain volume analysis (*n* = 1425) actually had a slightly smaller sample than the cognitive function analyses (*n* = 1513 to 1581) and the infarct and other SVD analyses (*n* = 1459 to 1464) after QC and exclusion of individuals with consistently fast or slow aging (Figure 2). Second, attenuation in *Model 2* could be due to measurement properties of the outcomes: cognitive function was assessed by questionnaires, which are prone to measurement error (diluting associations), and brain infarcts and other SVD are binary events for which subgrouping can leave too few events to retain statistical power. Third, unmeasured or residual confounding is unlikely to be an explanation, because both *Model 1* (full dataset) and *Model 2* (excluding consistent fast/slow agers) were adjusted for the same set of covariates; any such confounding would be expected to affect both models similarly.

Longitudinal MRI studies demonstrate that structural brain volume can change over relatively short intervals. In the Baltimore Longitudinal Study of Aging (BLSA) study cohort, GM and WM volumes declined by 1.32–2.76 cm^3^/year and 2.40–3.29 cm^3^/year, respectively, over 2 to 4 years among nondemented adults.^36^ Similarly, TBV decreased by ≈0.4%–0.5% per year in healthy adults across midlife and older age.^37^ These findings support our observation that shifts in the pace of aging over ≈5 years were associated with brain volume. Consistently, mediation analyses showed that midlife cardiovascular health was associated with late‐life brain volume, with DDPACE mediating 15%–17.7% of these associations (Figure 3A).

In contrast, downstream outcomes such as cognitive decline, incident infarcts, and other markers of SVD may require longer follow‐up to manifest, and the observed relationships may be driven primarily by enduring influences of earlier life‐course exposures, rather than by short‐term changes in the pace of aging. There is well‐established evidence linking early‐life lifestyle and environmental factors to cognitive function in later life. For example, individuals who experienced three to four adverse childhood events had a higher odds of developing poor psychomotor speed in old age (OR = 1.39, 95% CI: 1.00–1.93), with an even stronger association observed among those with five or more events (OR = 1.52, 95% CI: 1.07–2.17).^38^ Our mediation analysis also suggested that DDPACE accounted for 16.9% to 25.1% of the association between midlife cardiovascular risk factors and late‐life cognitive function (Figure 3B). However, because DDPACE was measured at baseline, it may reflect concurrent or cumulative biological processes rather than preceding brain atrophy, which develops over decades, and therefore may not represent a strictly antecedent mediator.

Taken together, although DDPACE was associated with brain pathology in the full sample, the attenuation of associations in the shift‐based analyses may point to critical periods of neurodegeneration earlier in the life course, during which cumulative damage occurs and may be less amenable to reversal, even when improvements in BA are observed in late life.

Our findings have important implications for population‐level risk stratification and future research on brain damage. Specifically, our results suggest that measuring BA using DDPACE may improve the identification of subgroups at higher risk of adverse brain health outcomes at the population level. The midlife analyses further indicate that a composite of healthy behaviors is associated with later‐life DDPACE and brain health outcomes up to 30 years later, highlighting its relevance for population‐based prevention strategies. This suggests that, when evaluating a generalized BA measure such as DDPACE, outcome measures may need to capture broader biological systems or multidimensional patterns of brain health rather than relying solely on single outcomes. Future studies of BA could clarify causal mechanisms and optimal intervention timing to reduce brain atrophy and dementia incidence.

## 5 STRENGTHS AND LIMITATIONS

This study has several strengths. We used a prospective design with baseline DDPACE and follow‐up assessment of multiple brain health domains, supporting temporal inference. By examining longitudinal changes in DDPACE and focusing on individuals with marked acceleration or deceleration, we applied a targeted, shift‐based approach to evaluate associations with brain atrophy. In addition, mediation analyses demonstrated that DDPACE partially mediates associations between midlife composite score of cardiovascular risk factors and late‐life brain health, providing insight into biological pathways linking modifiable midlife exposures to brain aging.

Limitations include the use of a single cohort, which may limit generalizability, although internal validation supported the robustness of findings. DDPACE was derived from peripheral blood DNA methylation and may not fully reflect brain‐specific aging processes. Incorporating brain MRI‐based BA metrics in future work could further strengthen the robustness and clinical relevance of our findings. The predominantly European ancestry of the cohort limits applicability to other populations, and dementia analyses were restricted to individuals who survived long enough for diagnosis, although analyses accounting for competing risk of death produced similar findings. Furthermore, the results of the shift‐based analysis should be interpreted in light of the exclusion of individuals at the two extreme ends of the aging spectrum, namely the consistently fast–fast group, which exhibited less favorable baseline brain health indicators, and the slow–slow group, which demonstrated comparatively more favorable brain health profiles.

## 6 CONCLUSION

Higher baseline DDPACE score was associated with diminished cognitive function, reduced brain volumes, and an increased likelihood of brain infarcts at follow‐up. When focusing on individuals with a substantial change in DDPACE during follow‐up, the association persisted for brain volume but not for cognitive function and brain infarcts and other SVD, suggesting that DDPACE may also capture the long‐term influence of lifestyle and environmental factors on brain health. Supporting this, we found that DDPACE mediates the association of midlife cardiovascular health with late‐life brain deterioration, providing some insight into the systemic factors, and not just one risk factor, leading to dementia and underlying pathologies. Furthermore, these findings underscore the potential of DDPACE to not only capture cumulative lifestyle and environmental influences on brain aging but also to identify individuals at elevated risk of dementia when their pace of aging accelerates.

## CONFLICT OF INTEREST STATEMENT

The authors declare no conflict of interest that affect the results of this research project. Author disclosures are available in the Supporting Information.

## CONSENT

Data were collected from human participants. All participants signed an informed consent, and ethical approval was granted by the Iceland National Bioethics Committee (VSN: 00‐063).

## Supporting information



Supporting Information

Supporting Information

Supporting Information

Supporting Information

## Data Availability

Study data may be accessed by qualified researchers following submission of a reasonable request. Data access applications should be directed to the Principal Investigator of AGES‐RS cohort, V. G. Guðnason (v.gudnason@hjarta.is). The R scripts used for the analyses are available on GitHub at https://github.com/niguurayugenetics/DunedinPACE_Brain‐pathologies_manunscript
