# Supplementary material for: Changes in DNA methylation–based aging predicts brain damage and dementia and reflects life‐course cardiovascular risk
Source: Alzheimers Dement. 2026 Jun 27;22(7):e71632. doi: 10.1002/alz.71632 (PMC13309854; doi:10.1002/alz.71632)
Supplement: Supplementary file 1 — Supporting Information [file ALZ-22-e71632-s002.docx]

**Supplementary File 1. Description of DNA methylation assays, MRI scans, cognitive function assessments, and dementia diagnoses.**

### **DNA methylation assay**

Whole blood-derived DNA samples from n=2,602 baseline participants and n=2,081 follow-up participants of the AGES-RS cohort were processed using a unified pipeline at the Huge-F facility (http://glimdna.org/) at Erasmus Medical Center. The samples were bisulfite treated using the Zymo EZ-96 DNA Methylation Kit (Zymo Research, Irvine, CA, USA), and DNA methylation levels were analyzed using Illumina's Infinium MethylEPIC v1 Manifest B5 platform, covering 865,918 CpGs. The BeadChip intensity data were then processed to obtain β-values. Before the estimation of epigenetic clocks, the raw beta values underwent Quantile Normalization. Quality control steps involved the exclusion of: (i) probes with a detection p-value > 0.01 in more than 10% of samples (n = 1,851), (ii) individuals with a probe detection p-value ≥ 0.01 (n = 9), (iii) non-CpG probes (n = 22,525), (iv) probes with fewer than 3 beads (n = 1,258), and (v) cross-reactive probes (n = 43,254). After quality control, 819,802 probes from autosomal chromosomes were retained for analysis. A total of 2,602 participants were included at baseline, of whom 2,081 also completed the second assessment.

### **MRI acquisition, brain tissue segmentation, and brain infarcts**

Details of magnetic resonance imaging (MRI) acquisition and brain volume measurements are discussed in detail by Muller et al and Sigurdsson et al.^1,2^ Briefly, brain MRI scans were performed using a 1.5-T Signa Twinspeed EXCITE system (GE Medical Systems, Waukesha, WI) as part of the AGES–Reykjavik Study. The imaging protocol included a proton density/T2-weighted fast spin-echo sequence (TE1 = 22 ms, TE2 = 90 ms, TR = 3220 ms, flip angle = 90°, FOV = 220 mm, matrix = 256×256, slice thickness = 3 mm); a T1-weighted 3D spoiled gradient echo sequence (TE = 8 ms, TR = 21 ms, flip angle = 30°, FOV = 240 mm, matrix = 256×256, slice thickness = 1.5 mm); a T2-weighted gradient-echo echo-planar imaging sequence (TE = 50 ms, TR = 3050 ms, flip angle = 90°, FOV = 220 mm, matrix = 256×256, slice thickness = 3 mm); and a fluid-attenuated inversion recovery (FLAIR) sequence (TE = 100 ms, TR = 8000 ms, inversion time = 2000 ms, flip angle = 90°, FOV = 220 mm, matrix = 256×256). These multispectral images enabled detailed analysis of brain tissue volumes, including gray matter (GM), white matter (WM), cerebrospinal fluid (CSF), and white matter lesions (WML).

Image processing and tissue segmentation were performed using a fully automated, high-throughput pipeline adapted from the Montreal Neurological Institute (MNI) framework and optimized for AGES-RS.^2^ T1-weighted images were first corrected for signal nonuniformity and then aligned to the ICBM152 template using affine stereotaxic registration. Other sequences were co-registered to the T1-weighted image to ensure spatial alignment. Signal intensity normalization across sequences was applied, followed by tissue classification using an artificial neural network in a four-dimensional intensity space (T1, T2, PD, and FLAIR). Volumes of GM, WM, CSF, and WML were calculated in native space using scale factors from the registration process. Total brain (TB) volume was defined as the sum of GM, WM, and WML, while intracranial volume (ICV) included TB and CSF volumes. In this study, we used relative measures of GM, WM, and WML, each calculated by dividing the respective volume by the ICV.

Brain parenchymal defects (infarcts) were areas of brain tissue damage exhibiting signal intensity similar to cerebrospinal fluid (CSF) on T2-weighted, FLAIR, and proton density-weighted images.^3^ Cortical infarct-like lesions were infarcts involving or confined to the cortical ribbon and surrounded by a hyperintense rim on FLAIR images. Subcortical infarct-like lesions were found deeper in the brain, without extending into the cortex, but similarly showed a surrounding bright signal on FLAIR. These lesions had a minimum diameter of 4 mm.

If a subcortical lesion lacked this surrounding bright signal on FLAIR and showed no signs of bleeding (no hemosiderin) on T2-weighted GRE-EPI images, it was classified as a large Virchow-Robin space (VRS), a normal variant, and excluded from the definition of subcortical infarcts in this analysis. No minimum size threshold was applied for infarcts located in the cerebellum. When a lesion spanned multiple brain regions, it was classified based on the region where the lesion was widest, regardless of its shape or orientation.^3^

### **Cognitive functions**

In this study, we assessed three cognitive domains: memory, processing speed, and working memory (executive function). Details of the cognitive test battery and composite score construction are described in Saczynski et al.^4^ Briefly, the memory composite score was derived from the immediate- and delayed-recall components of the California Verbal Learning Test. Processing speed was assessed using the Digit Symbol Substitution Test, the Figure Comparison Test, and Parts I (word naming) and II (color naming) of the Stroop Test. Executive function was evaluated using the Digits Backward Test, the Spatial Working Memory Test from the Cambridge Neuropsychological Test Automated Battery, and Part III (word-color interference) of the Stroop Test. For each domain, composite scores were calculated by standardizing the raw scores from individual tests into z-scores (mean = 0, standard deviation = 1) and averaging the z-scores across tests within each domain. Finaly, global cognitive function score, was calculated as the average of these three domain scores.

### **Dementia**

Dementia were ascertained through a 3-step diagnostic process involving expert consensus. All participants initially completed the Digit Symbol Substitution Test (DSST) and the Mini-Mental State Examination (MMSE). Individuals who screened positive, defined as a score of <18 on the DSST or <24 on the MMSE, underwent a second, more detailed neuropsychological test battery. A subset of these individuals, who also screened positive on the Trail Making Test or the Rey Auditory Verbal Learning Test, were referred for a neurological examination. In addition, proxies for this latter group were interviewed to gather information on medical history as well as cognitive, functional, and social changes relevant to dementia diagnosis. A final consensus diagnosis was determined by a multidisciplinary panel comprising a Geriatrician, Neurologist, Neuropsychologist, and Neuroradiologist. Details of the diagnostic steps are provided by Sigurdsson et al.^2^ The median follow-up time for dementia diagnosis from baseline (2002–2006) to the end of the study in 2015 was 10.4 years (IQR: 4.2 years).

**References**

1. Muller M, Sigurdsson S, Kjartansson O, et al. Late-life brain volume: a life-course approach. The AGES-Reykjavik study. *Neurobiol Aging*. 2016;41:86-92. doi:10.1016/j.neurobiolaging.2016.02.012

2. Sigurdsson S, Aspelund T, Forsberg L, et al. Brain tissue volumes in the general population of the elderly: the AGES-Reykjavik study. *Neuroimage*. 2012;59(4):3862-3870. doi:10.1016/j.neuroimage.2011.11.024

3. Saczynski JS, Sigurdsson S, Jonsdottir MK, et al. Cerebral infarcts and cognitive performance: importance of location and number of infarcts. *Stroke*. 2009;40(3):677-682. doi:10.1161/STROKEAHA.108.530212

4. Saczynski JS, Jónsdóttir MK, Garcia ME, et al. Cognitive impairment: an increasingly important complication of type 2 diabetes: the age, gene/environment susceptibility--Reykjavik study. *Am J Epidemiol*. 2008;168(10):1132-1139. doi:10.1093/aje/kwn228
