# Supplementary material for: Changes in DNA methylation–based aging predicts brain damage and dementia and reflects life‐course cardiovascular risk
Source: Alzheimers Dement. 2026 Jun 27;22(7):e71632. doi: 10.1002/alz.71632 (PMC13309854; doi:10.1002/alz.71632)
Supplement: Supplementary file 2 — Supporting Information [file ALZ-22-e71632-s001.docx]

**
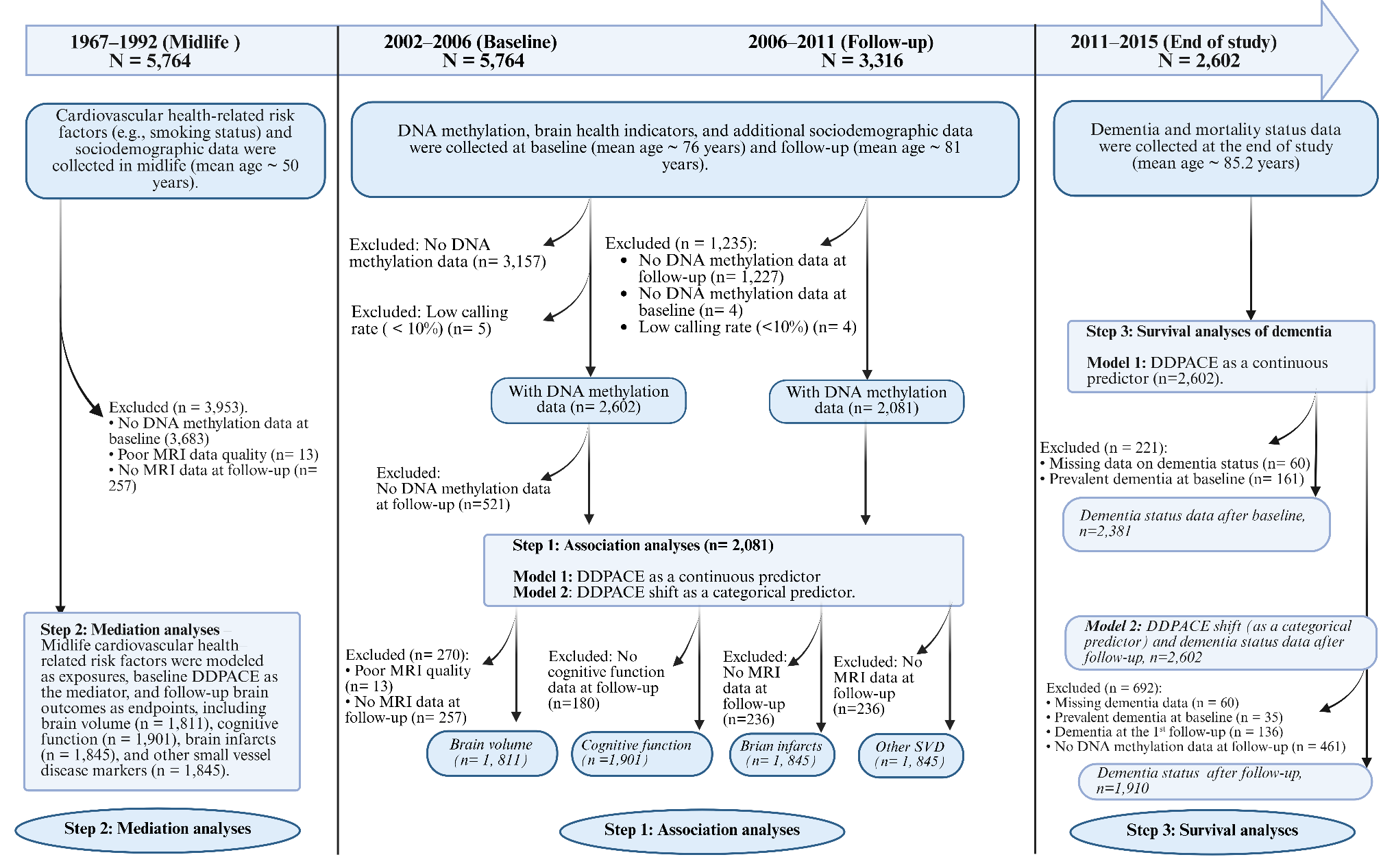
**

**Supplementary Figure 1. Flowchart showing the exclusion criteria and the number of participants included at each step of the analytical approach.**


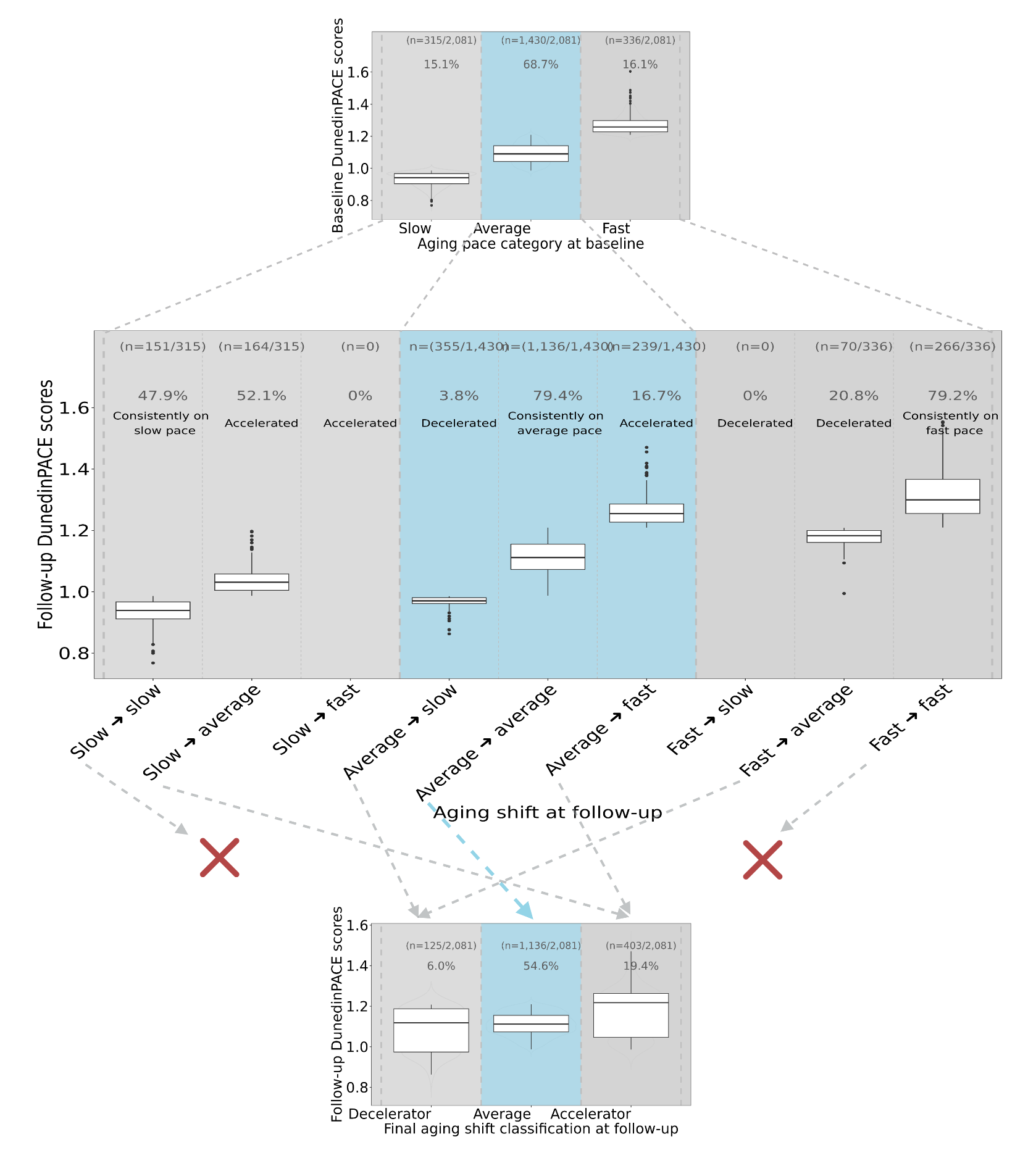


***Supplementary Figure 2. Pace of aging categories at baseline, shifts at follow-up assessment, and the final shift classification at follow-up.***

*This figure is adapted from our earlier work, which is currently under review.*

*Supplementary Figure 2 displays classification of participants into three aging pace categories based on the DunedinPACE score at baseline: Slow (≤ mean − 1 SD), Average (within ±1 SD of the mean), and Fast (≥ mean + 1 SD). The same thresholds from baseline were applied to the follow-up assessment. Participants with consistently “slow” or consistently “fast” aging profiles were excluded. The remaining participants were classified as “decelerators,” “average agers,” or “accelerators” based on changes in their aging pace between baseline and follow-up.*

**
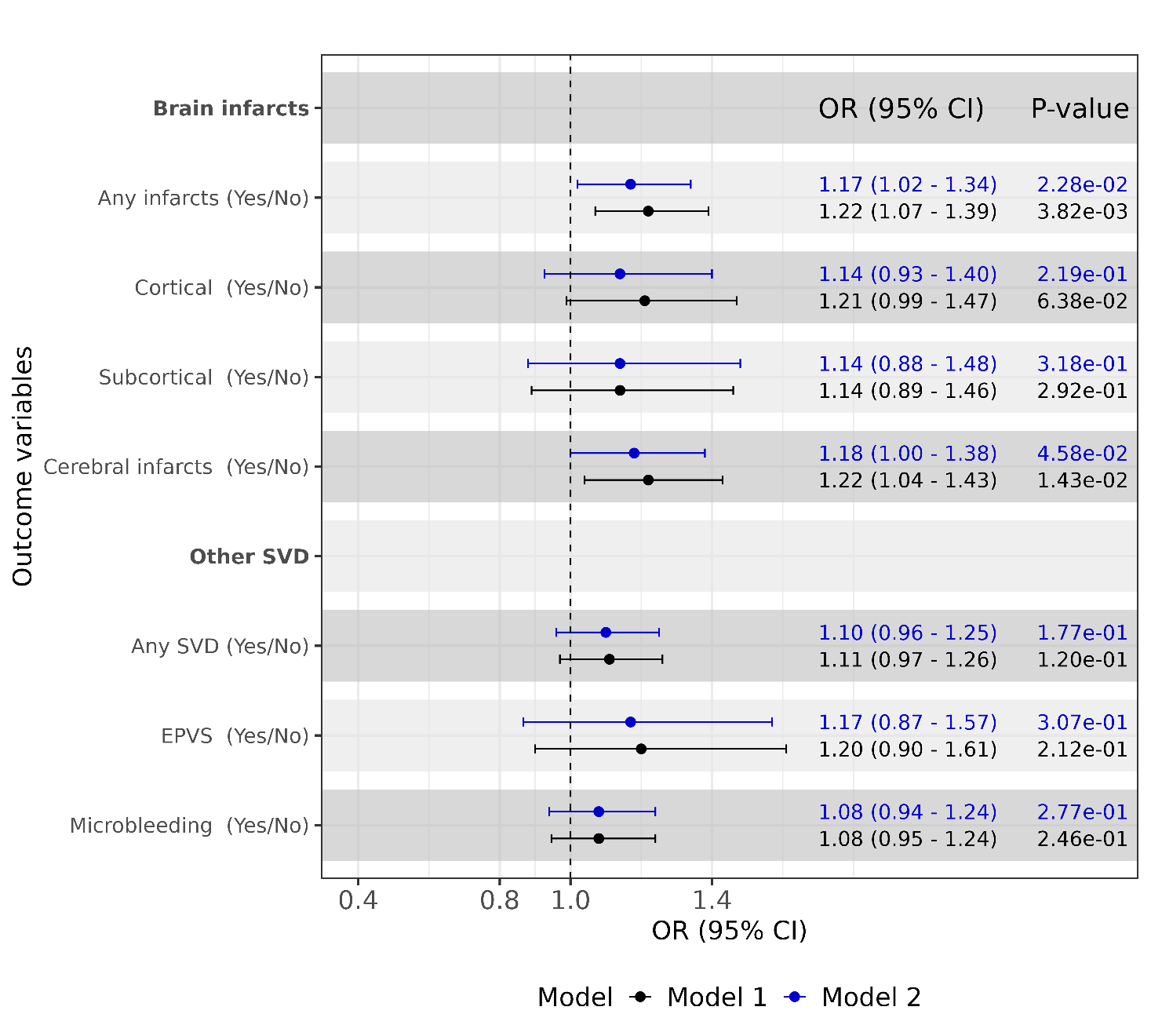
**

**Supplementary Figure 3. Association of DDPACE shifts with brain infarcts and other SVD at follow-up, before (*Model 1*) and after (*Model 2*) baseline values of respective outcome variables.** *All models were adjusted for chronological age, sex, smoking status, educational level, estimated white blood cell composition, and assay batch. Odds ratios (ORs) represent the odds of having infarcts or other SVD per 1 SD increase in DDPACE.*

*Abbreviation: DDPACE: DunedinPACE; SVD: Small vessel disease; EPVS: Enlarged perivascular spaces; OR: Odds ratio.*

**
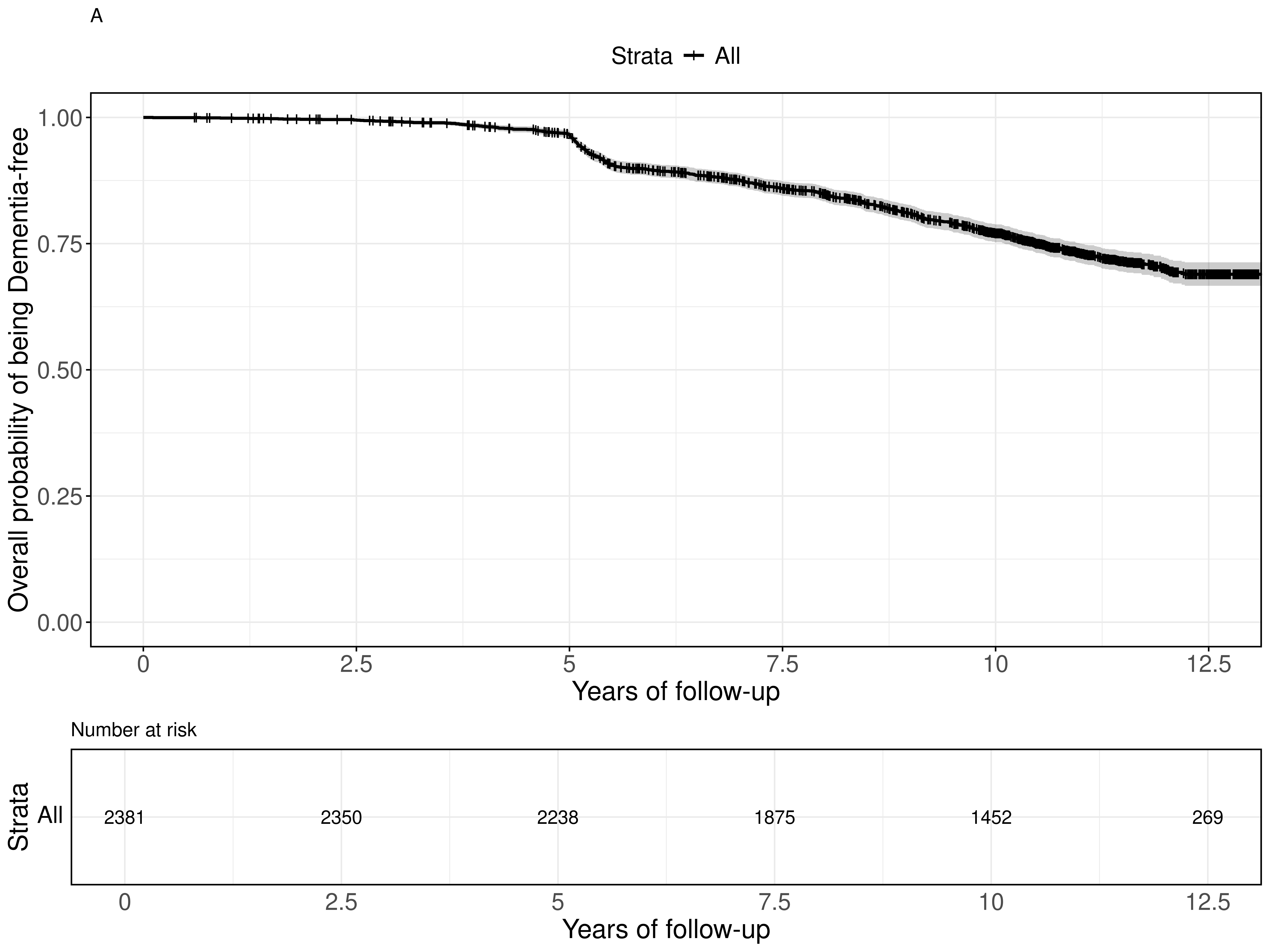
**

**Supplementary Figure 4A. Kaplan–Meier curve showing dementia-free probability for the overall sample.** *The follow-up period spanned from baseline (2002-2006) to the end of study (2011-2015).*

**
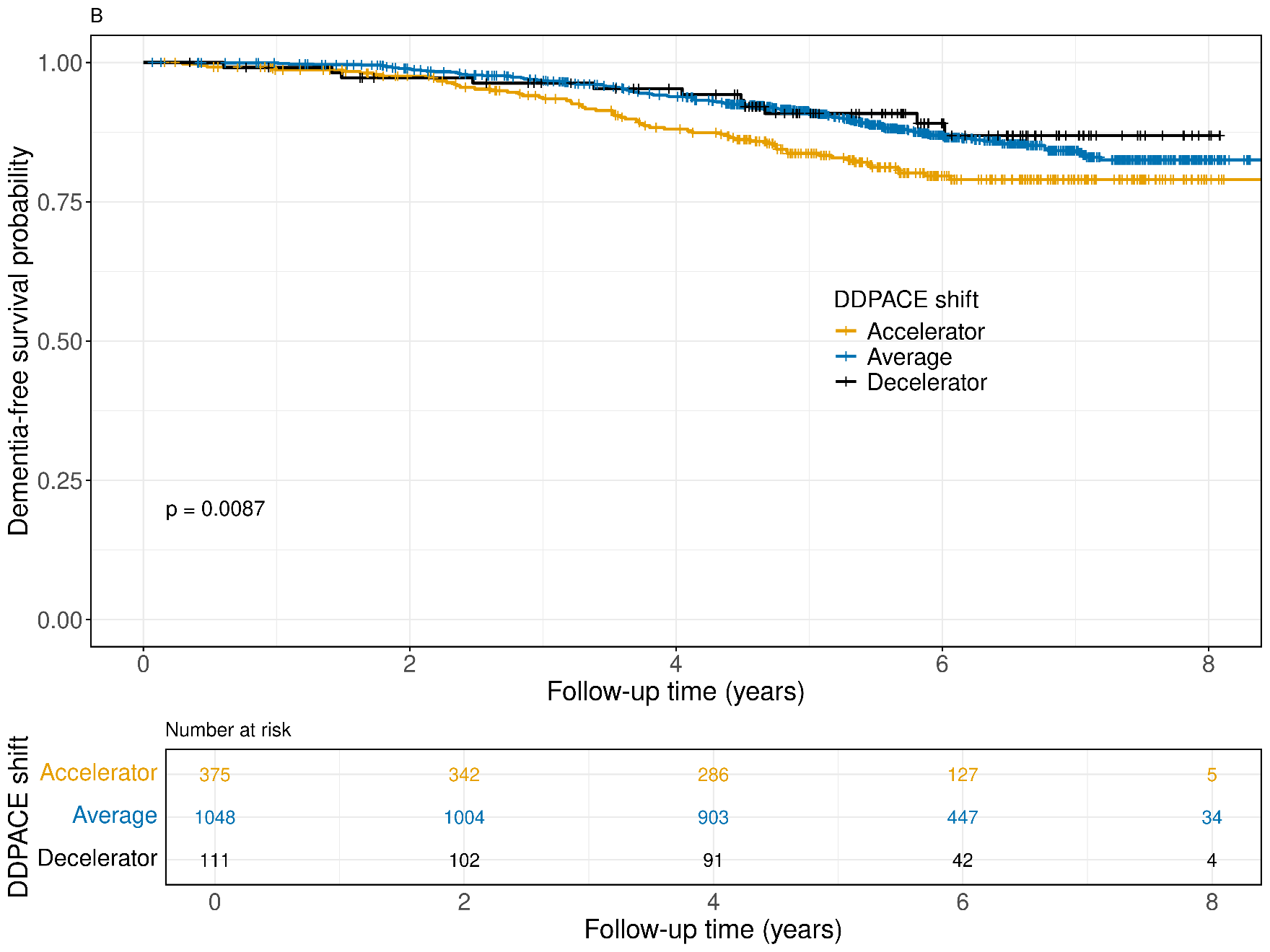
**

**Supplementary Figure 4B. Kaplan-Meier curves showing dementia-free probability by DunedinPACE shift categories.** *The follow-up period spanned from 2006–2011 to the end of study (2011–2015). See Supplementary Figure 2 for details on how individuals were categorized as decelerators, average agers, and accelerators based on changes in DDPACE between baseline and follow-up. Abbreviation: DDPACE: DunedinPACE.*
