# Supplementary material for: Changes in DNA methylation–based aging predicts brain damage and dementia and reflects life‐course cardiovascular risk
Source: Alzheimers Dement. 2026 Jun 27;22(7):e71632. doi: 10.1002/alz.71632 (PMC13309854; doi:10.1002/alz.71632)
Supplement: Supplementary file 3 — Supporting Information [file ALZ-22-e71632-s004.docx]

**Supplementary Table 1.** **Distribution of sociodemographic and cardiovascular health-related variables in Midlife.**

| Variable | Overall | Female | Male | p |
| --- | --- | --- | --- | --- |
| N | 2081 | 883 | 1198 |  |
| Age (years) (mean (SD)) | 50.12 (6.27) | 48.97 (5.81) | 50.96 (6.45) | <0.001 |
| Smoking status (%) |  |  |  |  |
| Never | 830 (39.9) | 224 (25.4) | 606 (50.6) | <0.001 |
| Former | 496 (23.8) | 252 (28.5) | 244 (20.4) |  |
| Current | 755 (36.3) | 407 (46.1) | 348 (29.0) |  |
| Physical activity (hours/week) (median [IQR]) | 0.05 [0.00, 1.50] | 0.05 [0.00, 1.50] | 0.05 [0.00, 1.50] | 0.993 |
| BMI (kg/m²) (mean (SD)) | 25.11 (3.47) | 25.45 (3.08) | 24.85 (3.71) | <0.001 |
| Systolic BP (mmHg) (mean (SD)) | 130.34 (15.71) | 133.46 (14.17) | 128.04 (16.39) | <0.001 |
| Diastolic BP (mmHg) (mean (SD)) | 82.65 (9.23) | 85.84 (8.88) | 80.30 (8.78) | <0.001 |
| Total cholesterol (mg/dL) (mean (SD)) | 6.30 (1.09) | 6.32 (1.01) | 6.29 (1.15) | 0.653 |
| Fasting blood glucose (mg/dL) (median [IQR]) | 77 (72.0, 83.0)] | 79 (74.0, 84.0) | 76 (71.0, 81.0) | <0.001 |
| Adapted-LS7 (mean (SD)) | 7.14 (1.84) | 6.57 (1.73) | 7.56 (1.80) | <0.001 |

**Supplementary Table 2A. Interaction of baseline DDPACE and APOE4 status in predicting cognitive function outcomes at follow-up.**

| Outcome | Predictor | Estimate | 95% CI lower | 95% CI upper | P-value |
| --- | --- | --- | --- | --- | --- |
| Memory | DDPACE (per 1 SD) | -0.03 | -0.07 | 0.01 | 1.70E-01 |
|  | APOE4 (Yes) | -0.18 | -0.25 | -0.11 | **4.70E-07** |
|  | DDPACE*APOE4 (Yes) | -0.01 | -0.08 | 0.06 | 6.92E-01 |
| Processing speed | DDPACE (per 1 SD) | -0.04 | -0.08 | -0.01 | **6.25E-03** |
|  | APOE4 (Yes) | -0.11 | -0.16 | -0.05 | **1.89E-04** |
|  | DDPACE*APOE4 (Yes) | -0.02 | -0.07 | 0.04 | 5.49E-01 |
| Working | DDPACE (per 1 SD) | -0.04 | -0.09 | 0.00 | **5.70E-02** |
|  | APOE4 (Yes) | -0.10 | -0.18 | -0.01 | **2.21E-02** |
|  | DDPACE*APOE4 (Yes) | -0.01 | -0.09 | 0.07 | 8.22E-01 |
| Global cognitive function | DDPACE (per 1 SD) | -0.04 | -0.07 | 0.00 | **2.54E-02** |
|  | APOE4 (Yes) | -0.15 | -0.21 | -0.09 | **4.67E-07** |
|  | DDPACE*APOE4 (Yes) | -0.01 | -0.07 | 0.05 | 6.36E-01 |

**Supplementary Table 2B. Association of baseline DunedinPACE with cognitive function outcomes at follow-up after excluding *APOE* ε2/ε4 carriers.**

|  | Per 1 SD increase in DDPACE | | | |
| --- | --- | --- | --- | --- |
| Outcome | **Estimate** | **95% CI lower** | **95% CI upper** | **P-value** |
| Memory | -0.03 | -0.07 | 0.00 | 8.55E-02 |
| Processing speed | -0.05 | -0.08 | -0.02 | **1.02E-03** |
| Working | -0.04 | -0.09 | 0.00 | **3.46E-02** |
| Global cognitive function | -0.04 | -0.07 | -0.01 | **6.91E-03** |

*All models in Supplementary Table 2A and 2B were adjusted for chronological age, sex, smoking status, educational level, estimated white blood cell composition, APOE4 status, and assay batch. Beta effect sizes represent the change in outcome (in SD units) per 1 SD increase in DDPACE scores.*

*Abbreviations: APOE4: apolipoprotein E4 carrier status; DDPACE: DunedinPACE; SD: Standard deviation; 95% CI lower: lower 95% confidence interval; 95% CI upper: upper 95% confidence interval.*

**Supplementary Table 3.** **Model performance and effect estimates of DDPACE (per 1 SD increase) on brain volumes, cognitive function, brain infarcts, and other SVD before and after 10-fold cross-validation.**

| **Outcome** | **Model** | **R-squared** | **Estimate** | **Lower 95 CI** | **Upper 95% CI** | **P** |
| --- | --- | --- | --- | --- | --- | --- |
| **Brain volume** |  |  |  |  |  |  |
| WM | Original | 0.85 | -0.03 | -0.05 | -0.001 | **1.73E-02** |
|  | 10-Fold CV | 0.85 | -0.03 | -0.03 | -0.02 | **3.13E-02** |
| WML | Original | 0.91 | 0.001 | -0.02 | 0.02 | 9.57E-01 |
|  | 10-Fold CV | 0.91 | 0.001 | -0.01 | 0.01 | 7.14E-01 |
| GM | Original | 0.85 | -0.03 | -0.05 | -0.01 | **9.86E-03** |
|  | 10-Fold CV | 0.85 | -0.03 | -0.04 | -0.02 | **2.04E-02** |
| TBV | Original | 0.90 | -0.03 | -0.04 | -0.01 | **3.72E-03** |
|  | 10-Fold CV | 0.90 | -0.03 | -0.03 | -0.02 | **1.01E-02** |
| **Cognitive function** |  |  |  |  |  |  |
| Memory | Original | 0.58 | -0.03 | -0.06 | 0.001 | 8.11E-02 |
|  | 10-Fold CV | 0.58 | -0.03 | -0.04 | -0.02 | 1.14E-01 |
| Processing speed | Original | 0.71 | -0.05 | -0.08 | -0.02 | **8.72E-04** |
|  | 10-Fold CV | 0.71 | -0.05 | -0.06 | -0.04 | **2.20E-03** |
| Working | Original | 0.41 | -0.05 | -0.09 | -0.01 | **2.43E-02** |
|  | 10-Fold CV | 0.42 | -0.05 | -0.06 | -0.03 | **4.06E-02** |
| Global cognitive function | Original | 0.69 | -0.04 | -0.07 | -0.01 | **5.91E-03** |
|  | 10-Fold CV | 0.70 | -0.04 | -0.05 | -0.03 | **1.03E-02** |
| **Brain infarcts** |  | **AUC** | **OR** | **Lower 95 CI** | **Upper 95% CI** | **P** |
| Infarcts (Yes/No) | Original | 0.72 | 1.17 | 1.02 | 1.34 | **2.28E-02** |
|  | 10-Fold CV | 0.65 | 1.17 | 1.14 | 1.21 | **3.48E-02** |
| **No. of infarcts** |  |  |  |  |  |  |
| None | Reference |  |  |  |  |  |
| One infarct | Original | 0.70 | 1.15 | 0.98 | 1.36 | 9.31E-02 |
|  | 10-Fold CV | 0.59 | 1.15 | 1.11 | 1.20 | 1.19E-01 |
| Two or more infarcts | Original | 0.79 | 1.18 | 0.96 | 1.45 | 1.09E-01 |
|  | 10-Fold CV | 0.67 | 1.18 | 1.14 | 1.23 | 1.36E-01 |
| **Other SVD** |  |  |  |  |  |  |
| Any other SVD (Yes/No) | Original | 0.67 | 1.10 | 0.96 | 1.25 | 1.77E-01 |
|  | 10-Fold CV | 0.58 | 1.10 | 1.06 | 1.14 | 2.14E-01 |
| **No. of SVD** |  |  |  |  |  |  |
| None | Reference |  |  |  |  |  |
| One SVD | Original | 0.65 | 1.10 | 0.93 | 1.25 | 3.41E-01 |
|  | 10-Fold CV | 0.51 | 1.08 | 1.01 | 1.15 | 3.92E-01 |
| Two or more SVD | Original | 0.80 | 1.10 | 0.86 | 1.37 | 4.80E-01 |
|  | 10-Fold CV | 0.67 | 1.10 | 1.01 | 1.17 | 5.33E-01 |

*All models were adjusted for chronological age, sex, smoking status, educational level, estimated white blood cell composition, and assay batch. Brain volume models were additionally adjusted for intracranial volume, while cognitive function models were further adjusted for APOE4 status. Beta estimates and ORs represent a change in outcome per 1 SD increase in DDPACE scores.*

*Abbreviations: DDPACE: DunedinPACE scores; SD: Standard deviation; WM: White matter; WML: White matter lesions; GM, Grey matter; TBV: Total brain volume; SVD: Small vessel disease; OR: odds ratios; CI: Confidence interval; P: P-value; CV: Cross validation.*

**Supplementary Table 4. Associations between change in DunedinPACE (ΔDDPACE; follow-up minus baseline) and brain health outcomes at follow-up, including brain volume measures, cognitive performance, and brain infarcts, in the full sample.**

| **Outcome** | **Estimate (95% CI)** | **P-value** | **N** |
| --- | --- | --- | --- |
| **Brain volume**^♀^ |  |  |  |
| WM | -0.054 (-0.098 - -0.011) | **1.51E-02** | 1816 |
| LogWML | 0.047 (0.001 - 0.093) | **4.49E-02** | 1816 |
| GM | -0.042 (-0.083 - -0.002) | **4.02E-02** | 1816 |
| TBV | -0.047 (-0.087 - -0.006) | **2.35E-02** | 1816 |
| **Cognitive function**^*^ |  |  |  |
| Memory | -0.039 (-0.080 - 0.002) | 6.36E-02 | 1891 |
| Processing speed | -0.037 (-0.076 - 0.002) | 6.61E-02 | 1972 |
| Working memory | -0.039 (-0.083 - 0.006) | 8.81E-02 | 1904 |
| Global cognitive function | -0.046 (-0.086 - -0.006) | **2.52E-02** | 1794 |
| **Brain infarcts or SVD** | **OR (95% CI)** | **P-value** | **Yes/Total** |
| Any infarct ("No" as reference) | 1.166 (1.035 - 1.314) | **1.17E-02** | 399/1837 |
| Any SVD ("No" as reference) | 1.053 (0.934 - 1.186) | 3.96E-01 | 402/1831 |

^♀^We used relative measures of GM, WM, and WML, each calculated by dividing the respective volume by the intracranial volume (head size); as such, these variables are unitless.

^*^Memory, processing speed, and working memory were derived as averages of standardized (z-scored) composites from multiple test tools (see *Supplementary File 1*).

*Abbreviations: WM: White matter; logWML: log-transformed White matter lesions; GM, Grey matter; TBV: Total brain volume; SVD: Small vessel disease; OR: Odds ratio;* *95%CI: 95% Confidence interval; P: P-value.*

**Supplementary Table 5A.** **Associations of residuals of baseline epigenetic clocks (Horvath, Hannum, PhenoAge, and GrimAge) with brain volume and cognitive function outcomes at follow-up.**

|  |  | Before adjusting for the baseline values of the outcomes | | | | After adjusting for baseline values of the outcomes | | | |
| --- | --- | --- | --- | --- | --- | --- | --- | --- | --- |
| Clock | **Outcome** | **Estimate** | **Lower 95% CI** | **Upper 95% CI** | **P** | **Estimate** | **Lower 95% CI** | **Upper 95% CI** | **P** |
|  | **Brain volume** | |  |  |  |  |  |  |  |
| PC-Horvath | WM | -0.02 | -0.06 | 0.02 | 3.55E-01 | -0.01 | -0.03 | 0.01 | 3.24E-01 |
|  | WML | 0.04 | -0.01 | 0.08 | 1.09E-01 | -0.01 | -0.02 | 0.01 | 5.05E-01 |
|  | GM | -0.05 | -0.09 | -0.01 | **1.80E-02** | -0.01 | -0.03 | 0.01 | 2.98E-01 |
|  | TBV | -0.04 | -0.08 | 0.00 | 6.83E-02 | -0.01 | -0.03 | 0.00 | 1.71E-01 |
| PC-Hannum | WM | -0.04 | -0.09 | 0.01 | 8.77E-02 | -0.01 | -0.03 | 0.01 | 3.94E-01 |
|  | WML | 0.03 | -0.02 | 0.08 | 1.83E-01 | -0.02 | -0.04 | -0.01 | 5.95E-03 |
|  | GM | -0.03 | -0.07 | 0.02 | 2.27E-01 | -0.02 | -0.04 | 0.01 | 1.47E-01 |
|  | TBV | -0.03 | -0.08 | 0.01 | 1.30E-01 | -0.02 | -0.04 | 0.00 | **3.44E-02** |
| PC-PhenoAge | WM | -0.06 | -0.10 | -0.01 | **9.12E-03** | -0.01 | -0.03 | 0.01 | 3.56E-01 |
|  | WML | 0.04 | -0.01 | 0.09 | 8.19E-02 | 0.001 | -0.01 | 0.02 | 7.03E-01 |
|  | GM | -0.05 | -0.09 | -0.01 | **1.16E-02** | -0.01 | -0.02 | 0.01 | 6.00E-01 |
|  | TBV | -0.07 | -0.11 | -0.03 | **1.30E-03** | 0.00 | -0.02 | 0.01 | 6.35E-01 |
| PC-GrimAge | WM | -0.18 | -0.24 | -0.12 | **1.42E-08** | -0.04 | -0.07 | -0.01 | **2.53E-03** |
|  | WML | 0.08 | 0.02 | 0.15 | **1.34E-02** | 0.001 | -0.02 | 0.02 | 9.28E-01 |
|  | GM | -0.13 | -0.19 | -0.08 | **5.85E-06** | -0.001 | -0.03 | 0.02 | 8.04E-01 |
|  | TBV | -0.18 | -0.24 | -0.12 | **6.21E-10** | -0.01 | -0.03 | 0.01 | 3.32E-01 |
|  | **Cognitive function** | |  |  |  |  |  |  |  |
| PC-Horvath | Memory | -0.01 | -0.06 | 0.03 | 4.71E-01 | 0.01 | -0.03 | 0.04 | 7.14E-01 |
|  | Processing speed | -0.001 | -0.04 | 0.04 | 8.37E-01 | 0.00 | -0.02 | 0.03 | 9.04E-01 |
|  | Working | -0.001 | -0.04 | 0.05 | 9.21E-01 | 0.00 | -0.04 | 0.03 | 8.16E-01 |
| PC-Hannum | Memory | -0.03 | -0.08 | 0.01 | 1.28E-01 | -0.01 | -0.04 | 0.02 | 5.55E-01 |
|  | Processing speed | -0.03 | -0.07 | 0.02 | 2.31E-01 | -0.01 | -0.04 | 0.02 | 3.82E-01 |
|  | Working | -0.01 | -0.06 | 0.04 | 7.14E-01 | -0.001 | -0.04 | 0.04 | 8.11E-01 |
| PC-PhenoAge | Memory | -0.02 | -0.07 | 0.02 | 2.41E-01 | 0.01 | -0.03 | 0.04 | 7.29E-01 |
|  | Processing speed | -0.02 | -0.06 | 0.02 | 2.92E-01 | -0.02 | -0.04 | 0.01 | 2.38E-01 |
|  | Working | -0.01 | -0.05 | 0.04 | 7.53E-01 | -0.001 | -0.04 | 0.04 | 9.94E-01 |
| PC-GrimAge | Memory | -0.07 | -0.13 | -0.01 | **1.76E-02** | -0.03 | -0.08 | 0.01 | 1.28E-01 |
|  | Processing speed | -0.11 | -0.16 | -0.05 | **1.04E-04** | -0.04 | -0.08 | -0.01 | **2.28E-02** |
|  | Working | -0.09 | -0.16 | -0.03 | **3.10E-03** | -0.05 | -0.10 | 0.00 | **4.97E-02** |

**Supplementary Table 5B. Associations of residuals of baseline epigenetic clocks (Horvath, Hannum, PhenoAge, and GrimAge) with brain infarct outcomes at follow-up.**

| Clock | Outcome | Before adjusting for baseline values of the outcomes | | | | After adjusting for baseline values of the outcomes | | | | |
| --- | --- | --- | --- | --- | --- | --- | --- | --- | --- | --- |
|  | **Brain infarcts** | **OR** | **Lower 95% CI** | **Upper 95% CI** | **P** | **OR** | **Lower 95% CI** | **Upper 95% CI** | **P** |  |
| PC-Horvath | Any infarcts (Yes/No) | 1.00 | 0.89 | 1.13 | 9.56E-01 | 1.01 | 0.89 | 1.14 | 8.92E-01 |  |
|  | Cortical (Yes/No) | 0.86 | 0.72 | 1.03 | 1.03E-01 | 0.86 | 0.71 | 1.03 | 1.02E-01 |  |
|  | Subcortical (Yes/No) | 1.01 | 0.81 | 1.24 | 9.63E-01 | 0.97 | 0.77 | 1.20 | 7.58E-01 |  |
|  | Cerebral infarcts (Yes/No) | 1.05 | 0.91 | 1.21 | 5.00E-01 | 1.05 | 0.90 | 1.21 | 5.28E-01 |  |
|  | EPVS (Yes/No) | 1.21 | 0.94 | 1.54 | 1.22E-01 | 1.23 | 0.95 | 1.57 | 1.06E-01 |  |
|  | Micro bleeding (Yes/No) | 1.08 | 0.96 | 1.22 | 2.20E-01 | 1.07 | 0.94 | 1.21 | 2.96E-01 |  |
| PC-Hannum | Any infarcts (Yes/No) | 1.00 | 0.88 | 1.13 | 9.97E-01 | 1.01 | 0.89 | 1.15 | 8.51E-01 |  |
|  | Cortical (Yes/No) | 0.97 | 0.81 | 1.17 | 7.80E-01 | 1.01 | 0.83 | 1.22 | 9.07E-01 |  |
|  | Subcortical (Yes/No) | 0.94 | 0.74 | 1.20 | 6.47E-01 | 0.92 | 0.71 | 1.17 | 4.95E-01 |  |
|  | Cerebral infarcts (Yes/No) | 0.94 | 0.80 | 1.09 | 3.93E-01 | 0.93 | 0.79 | 1.09 | 3.71E-01 |  |
|  | EPVS (Yes/No) | 1.30 | 1.00 | 1.66 | **4.08E-02** | 1.24 | 0.96 | 1.60 | 9.61E-02 |  |
|  | Micro bleeding (Yes/No) | 1.09 | 0.96 | 1.24 | 1.78E-01 | 1.09 | 0.96 | 1.25 | 1.85E-01 |  |
| PC-PhenoAge | Any infarcts (Yes/No) | 1.08 | 0.96 | 1.22 | 1.90E-01 | 1.07 | 0.94 | 1.21 | 2.94E-01 |  |
|  | Cortical (Yes/No) | 1.06 | 0.89 | 1.25 | 5.23E-01 | 1.03 | 0.86 | 1.23 | 7.74E-01 |  |
|  | Subcortical (Yes/No) | 1.03 | 0.83 | 1.27 | 7.68E-01 | 1.01 | 0.81 | 1.25 | 9.05E-01 |  |
|  | Cerebral infarcts (Yes/No) | 1.12 | 0.97 | 1.28 | 1.14E-01 | 1.09 | 0.95 | 1.26 | 2.26E-01 |  |
|  | EPVS (Yes/No) | 1.21 | 0.95 | 1.54 | 1.16E-01 | 1.24 | 0.96 | 1.57 | 8.84E-02 |  |
|  | Micro bleeding (Yes/No) | 1.05 | 0.93 | 1.18 | 4.60E-01 | 1.04 | 0.92 | 1.17 | 5.72E-01 |  |
| PC-GrimAge | Any infarcts (Yes/No) | 1.21 | 1.03 | 1.44 | **2.31E-02** | 1.17 | 0.99 | 1.39 | 7.04E-02 |  |
|  | Cortical (Yes/No) | 1.12 | 0.88 | 1.44 | 3.59E-01 | 1.09 | 0.84 | 1.40 | 5.30E-01 |  |
|  | Subcortical (Yes/No) | 0.88 | 0.64 | 1.20 | 4.29E-01 | 0.92 | 0.66 | 1.26 | 5.98E-01 |  |
|  | Cerebral infarcts (Yes/No) | 1.29 | 1.06 | 1.58 | **1.26E-02** | 1.26 | 1.02 | 1.54 | **2.99E-02** |  |
|  | EPVS (Yes/No) | 1.36 | 0.95 | 1.94 | 9.18E-02 | 1.30 | 0.91 | 1.85 | 1.54E-01 |  |
|  | Micro bleeding (Yes/No) | 1.06 | 0.89 | 1.26 | 4.85E-01 | 1.07 | 0.90 | 1.27 | 4.63E-01 |  |
|  | **No. of total counts** |  |  |  |  |  |  |  |  |  |
|  | None | Reference | |  |  |  |  |  |  |  |
| PC-Horvath | One infarct of any type | 1.08 | 0.934 | 1.24 | 3.05E-01 | 1.08 | 0.93 | 1.24 | 3.28E-01 |  |
|  | Two or more any infarcts | 0.891 | 0.744 | 1.07 | 2.08E-01 | 0.87 | 0.722 | 1.05 | 1.45E-01 |  |
|  | None | Reference |  |  |  |  |  |  |  |  |
| PC-Hannum | One infarct of any type | 1.11 | 0.957 | 1.29 | 1.66E-01 | 1.11 | 0.953 | 1.29 | 1.79E-01 |  |
|  | Two or more any infarcts | 0.834 | 0.683 | 1.02 | 7.40E-02 | 0.831 | 0.676 | 1.02 | 8.00E-02 |  |
|  | None | Reference | |  |  |  |  |  |  |  |
| PC-PhenoAge | One infarct of any type | 1.06 | 0.918 | 1.23 | 4.21E-01 | 1.05 | 0.901 | 1.21 | 5.57E-01 |  |
|  | Two or more any infarcts | 1.12 | 0.943 | 1.32 | 1.99E-01 | 1.07 | 0.901 | 1.28 | 4.23E-01 |  |
|  | None | Reference | |  |  |  |  |  |  |  |
| PC-GrimAge | One infarct of any type | 1.18 | 0.955 | 1.45 | 1.27E-01 | 1.14 | 0.922 | 1.4 | 2.28E-01 |  |
|  | Two or more any infarcts | 1.25 | 0.983 | 1.6 | 6.87E-02 | 1.18 | 0.922 | 1.52 | 1.86E-01 |  |

*All models were adjusted for chronological age, sex, smoking status, educational level, estimated white blood cell composition, and assay batch. Brain volume models additionally adjusted for intracranial volume, while cognitive function models further adjusted for APOE4 status. Beta estimates and ORs represent a change in outcome per 1 SD increase in the residual of the corresponding epiclocks.*

*Abbreviations: PC: Principal components-based DNA methylation clocks; WM: White matter; WML: White matter lesions; GM, Grey matter; TBV: Total brain volume; SVD: Small vessel disease; EPVS: Enlarged perivascular spaces; SD: Standard deviation; OR: Odds ratios; CI: Confidence interval; P: P-value.*

**Supplementary Table 6. Association between baseline DDPACE and incident dementia during follow-up: (1) after excluding individuals with the APOE ε2/ε4 genotype; (2) applying a 2-year lag period; and (3) interaction analyses of baseline DDPACE and DDPACE shift with APOE4 status, sex, smoking status, and educational level over the full follow-up period.**

|  | **Variable** | **HR (95% CI)** | **P** | **n/N*** | **Schoenfeld’s**  **P** |
| --- | --- | --- | --- | --- | --- |
| **Baseline DDPACE scores (continuous)** | | |  |  |  |
| 1. **After excluding individuals with ε2/ε4 genotype (n=37)** | |  |  |  |  |
|  | Per 1 SD increase of baseline DDPACE scores | 1.21 (1.07-1.37) | **1.79E-03** | 377/1,986 | 5.9E-02 |
| 1. **Two-year lag time** | |  |  |  |  |
|  | Per 1 SD increase of baseline DDPACE scores | 1.21 (1.07-1.36) | **2.13E-03** | 383/2,023 | 1.48E-01 |
| 1. **Interaction analyses of DDPACE with sociodemographic and lifestyle factors** | |  |  |  |  |
| - 1. **Baseline DDPACE** | |  |  |  |  |
|  | **Interaction with APOE4 status** |  |  |  |  |
|  | Per 1 SD increase of baseline DDPACE scores | 1.26 (1.10 -1.45) | **1.04E- 3** | 383/2,023 | 1.21E-01 |
|  | APOE4 (Yes) | 2.05 (1.65 -2.55) | **7.42E-11** |  |  |
|  | Per 1 SD increase of baseline DDPACE scores*APOE4 (Yes) | 0.87 (0.69 -1.08) | 2.10E- 1 |  |  |
|  | **Interaction with sex** |  |  |  |  |
|  | Per 1 SD increase of baseline DDPACE scores | 1.20 (1.04 - 1.39) | **1.50E-02** | 383/2,023 | 6.0E-02 |
|  | Sex (M) | 0.96 (0.76 - 1.23) | 7.66E-01 |  |  |
|  | Per 1 SD increase of baseline DDPACE scores*Sex (M) | 1.01 (0.81 - 1.26) | 9.29E-01 |  |  |
|  | **Interaction with smoking status** |  |  |  |  |
|  | Per 1 SD increase of baseline DDPACE scores | 1.16 (0.98 - 1.38) | **7.64E-02** | 383/2,023 | 3.80E-01 |
|  | Never | Reference |  |  |  |
|  | Ex-smokers | 1.02 (0.81 - 1.28) | 8.77E-01 |  |  |
|  | Current smokers | 1.53 (1.01 - 2.32) | **4.55E-02** |  |  |
|  | Per 1 SD increase of baseline DDPACE scores*Ex-smokers | 1.13 (0.90 - 1.42) | 2.85E-01 |  |  |
|  | Per 1 SD increase of baseline DDPACE scores*Current smokers | 0.85 (0.59 - 1.22) | 3.80E-01 |  |  |
|  | **Interaction with educational level** |  |  |  |  |
|  | Per 1 SD increase of baseline DDPACE scores | 1.08 (0.87 - 1.35) | 4.84E-01 | 383/2,023 | 1.06E-01 |
|  | Elementary | Reference |  |  |  |
|  | High school | 0.97 (0.74 - 1.26) | 8.15E-01 |  |  |
|  | College | 1.03 (0.74 - 1.44) | 8.63E-01 |  |  |
|  | University | 0.94 (0.63 - 1.41) | 7.69E-01 |  |  |
|  | Per 1 SD increase of baseline DDPACE scores*High school | 1.20 (0.93 - 1.55) | 1.64E-01 |  |  |
|  | Per 1 SD increase of baseline DDPACE scores*College | 1.21 (0.85 - 1.71) | 2.91E-01 |  |  |
|  | Per 1 SD increase of baseline DDPACE scores*University | 0.87 (0.58 - 1.31) | 4.94E-01 |  |  |
| **3.2. DDPACE shifts (categorical)** | |  |  |  |  |
|  | **Interaction with sex** |  |  |  |  |
|  | Average agers | Reference |  | 204/1,525 | 7.75E-01 |
|  | Decelerators | 0.75 (0.29 - 1.92) | 5.48E-01 |  |  |
|  | Accelerators | 1.57 (1.06 - 2.33) | **2.40E-02** |  |  |
|  | Sex (M) | 0.76 (0.51 - 1.13) | 1.70E-01 |  |  |
|  | Decelerators* Sex (M) | 1.38 (0.38 - 4.97) | 6.23E-01 |  |  |
|  | Accelerators * Sex (M) | 1.06 (0.55 - 2.02) | 8.67E-01 |  |  |
|  | **Interaction with smoking status** |  |  |  |  |
|  | Average agers | Reference |  | 204/1,525 | 6.04E-01 |
|  | Decelerators | 0.60 (0.21 - 1.69) | 3.32E-01 |  |  |
|  | Accelerators | 1.49 (0.94 - 2.35) | 8.72E-02 |  |  |
|  | Ex-smokers | 1.12 (0.77 - 1.62) | 5.70E-01 |  |  |
|  | Current smokers | 1.08 (0.50 - 2.32) | 8.51E-01 |  |  |
|  | Decelerators * Ex-smokers | 2.03 (0.53 - 7.86) | 3.05E-01 |  |  |
|  | Accelerators * Ex-smokers | 0.99 (0.51 - 1.91) | 9.79E-01 |  |  |
|  | Decelerators * Current smokers | 2.23 (0.21 - 23.60) | 5.04E-01 |  |  |
|  | Accelerators * Current smokers | 2.27 (0.72 - 7.20) | 1.63E-01 |  |  |
|  | **Interaction with educational level** |  |  |  |  |
|  | Average agers | Reference |  | 204/1,525 | 5.98E-01 |
|  | Decelerators | 0.92 (0.46-1.85) | 8.11E-01 |  |  |
|  | Accelerators | 1.26 (0.80-1.98) | 3.12E-01 |  |  |
|  | High school | 1.02 (0.71-1.46) | 9.22E-01 |  |  |
|  | College | 0.95 (0.60-1.49) | 8.10E-01 |  |  |
|  | University | 0.94 (0.56-1.58) | 8.04E-01 |  |  |
|  | Decelerators * High school | 0.79 (0.33-1.90) | 6.03E-01 |  |  |
|  | Accelerators * High school | 0.95 (0.55-1.63) | 8.44E-01 |  |  |
|  | Decelerators * College | 0.89 (0.30-2.62) | 8.32E-01 |  |  |
|  | Accelerators * College | 1.25 (0.61-2.56) | 5.38E-01 |  |  |
|  | Decelerators * University | 0.78 (0.19-3.18) | 7.32E-01 |  |  |
|  | Accelerators * University | 1.01 (0.45-2.28) | 9.78E-01 |  |  |

*All models were adjusted for chronological age, sex, smoking status, educational level, estimated white blood cell composition, APOE4 status, and assay batch.*

*Abbreviations: DDPACE: DunedinPACE scores; SD: Standard deviation; HR: Hazard ratios; CI: Confidence interval; P: P-value; APOE4: Apolipoprotein E gene.*

*n/N*: Number of dementia cases per total number of observations used in the model.*
